# Quandong stones: A specialised Australian nut-cracking tool

**DOI:** 10.1371/journal.pone.0222680

**Published:** 2019-10-02

**Authors:** Colin Pardoe, Richard Fullagar, Elspeth Hayes

**Affiliations:** 1 Archaeology & Natural History, The Australian National University, Canberra, ACT, Australia; 2 Centre for Archaeological Science, School of Earth, Atmospheric and Life Sciences, University of Wollongong, Wollongong, NSW, Australia; Max Planck Institute for the Science of Human History, GERMANY

## Abstract

The quandong or native peach (*Santalum acuminatum* R.Br.) has been recognised as an important and tasty food resource among Aboriginal Australians in arid and semi-arid areas of southern Australia. It is valued for its fruit that is consumed raw or dried, and for its kernel, which is eaten raw or ground into paste for medicinal and skin care purposes. This paper reports on a study of ground stone implements within the Murray Darling Basin that has identified quandong stones as a distinct type of implement made specifically for the efficient cracking of quandong nuts. Data are presented on 1,327 ground stone implements from collections in 12 different locations in the Murray-Darling Basin (MDB), an area almost completely devoid of stone sources. Given the paucity of stone, multi-purpose use of implements is widely documented. Although it was common to find pits present in mortars and other ground stone tools demonstrating multiple functions, including use as anvils, a class of single purpose stones with multiple pits and distinctive form was identified. Most of these were found in areas known for groves of quandong and four were analysed for use-wear and residues along with two other ground stone items from the MDB. The results support their identification as specialised anvil stones for cracking quandong nuts.

## Introduction

Nuts from a wide range of species have formed an important human food resource for millennia across the world. Evidence of human nut cracking to access oil and protein rich kernels has been documented from stone implements found in Palaeolithic, Mesolithic and more recent archaeological sites in Europe and the Levant; from palaeobotanical data; from ethnographic accounts; and research into contemporary foraging activities in southern Africa [1–4]. Stone tools purportedly used for nut cracking have various names including *pitted stone cobbles*, *anvil* or *nutting* stones, pitted stone hammers and cupstones [1,3,5–8], and have provided some archaeological evidence for nut consumption. The tools commonly include a specialised lower (immobile) implement with a depression or pit to hold the hard-shelled nuts, which were cracked open with an upper (mobile) hand-held stone hammer. Archaeological evidence for nut cracking, though, has been the subject of debate. One school of thought identifies pits as the product of stone knapping and in particular bipolar reduction using a hammer and anvil [3,9]. An alternative hypothesis ‘views pits as the by-product of a particular task–the cracking of nuts’ [1: 2455]. Other mechanisms of pit formation have also been proposed and queried [1: 1588]. In Australia, Frederick McCarthy made the case for at least two functions. He had witnessed pits in anvil stones formed by hard woody seeds being broken on them by Aboriginal people using a stone hammer in Arnhem Land, northern Australia. He also suggested that pits were formed ‘by the pointed or conical (distal) end of a core placed on the anvil as a firm base for knapping flakes’ [10: 55]. This practice has been well documented within Australia both archaeologically and experimentally [11].

Experimental studies of stones used for cracking hazelnuts and hard seeds indicate that use-wear from stone knapping, nut cracking and kernel grinding can be distinct [1,12–15]. Our paper contributes further to this debate by distinguishing between a pitted stone implement designed specifically for large-scale processing of quandong nuts and pits recorded on a range of multi-purpose stones.

The multi-purpose nature of many Australian ground stone implements has hindered classification by type [16–19]. At the same time, however, this provides an opportunity to examine functions across a range of items, to investigate food-getting strategies, to build a better picture of the relationship between materials and their source, and to examine stone tool distribution and variety across different ecologies [13,14,20].

We describe the distribution of pits, one functional attribute of ground stone artefacts, across different collections in the Murray Darling Basin [MDB] in south-eastern Australia, and among different tool types. We use object morphology, spatial patterning, use-wear and residue analysis to set out a rationale and supporting evidence for considering some of these objects to be single purpose stones made for the sole purpose of cracking and processing quandong nuts.

**Fig 1:**
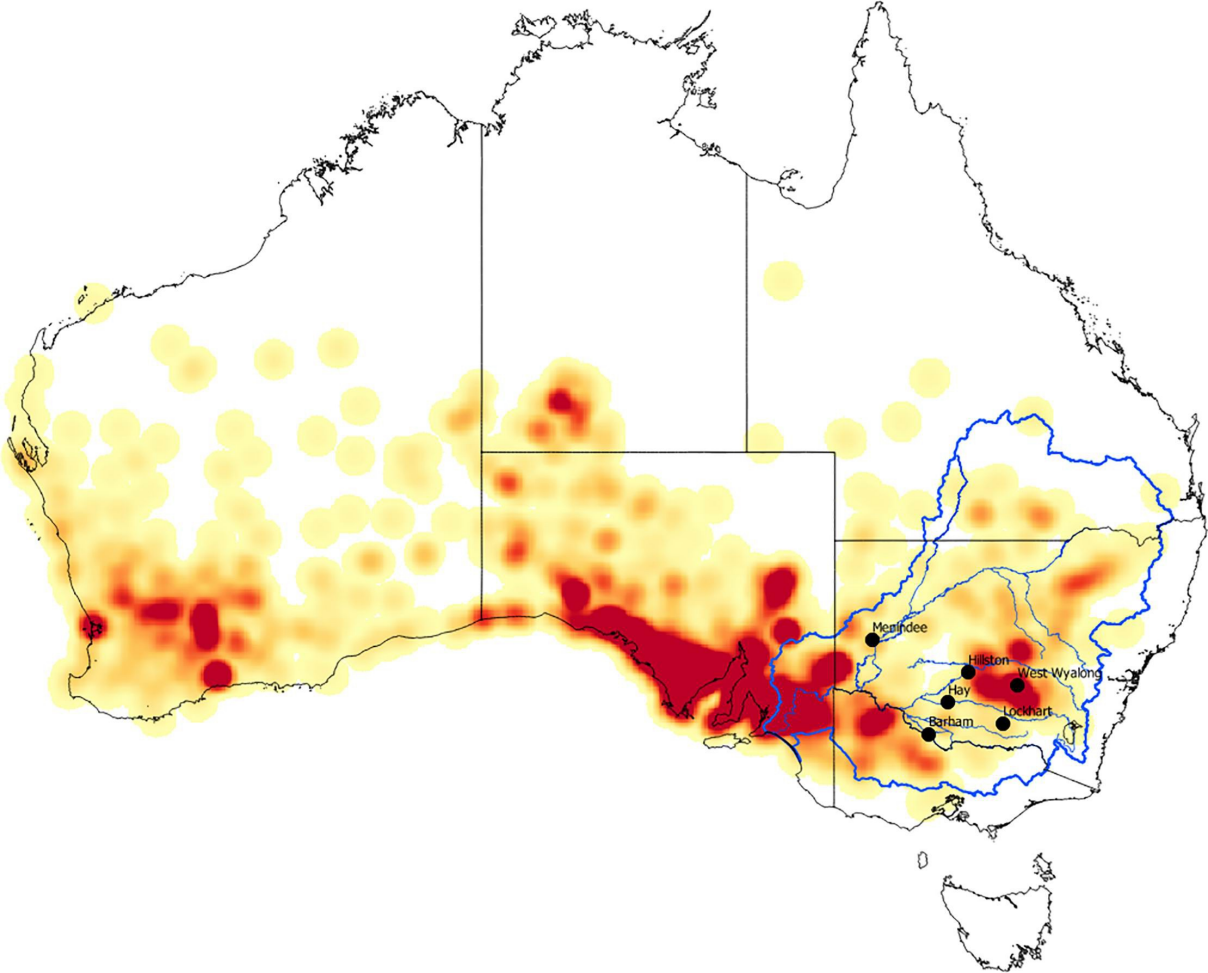
Quandong (*Santalum acuminatum*) distribution. The quandong (*Santalum acuminatum*) is distributed across the southern half of the Australian continent. Map created by CP using data from Geoscience Australia (Commonwealth of Australia (Geoscience Australia) 2012. This material is released under the Creative Commons Attribution 3.0 Australia Licence ‐ http://creativecommons.org/licenses/by/3.0/au/) and Atlas of living Australia (website accessed Nov 2018-11-21, https://www.ala.org.au/, ©OpenStreetMap contributors).

Our initial interest in what we have come to call a ‘quandong stone’ came about through shared interests in foraging strategies, spatial variation in ground stone assemblages, ethnographic observations, and traditional knowledge of activities that have not been widely recognised. Fruits and nuts traditionally comprised important plant foods across Australia. Quandongs, widely distributed across arid and semi-arid southern Australia (Fig 1), provide both fruit and kernels that can be processed and stored. They are the only large hard-shelled nut with an edible kernel in the MDB and have been identified ethnographically as a staple food and medicine [21,22,23,24].

The edible part on the outside, we’d gather and eat. And as for the stones–we’d collect them too, hit them with a rock, prise them open, and get the kernels,—you know, the inside part. We’d gather up a whole lot of them. My grandmother would gather them up and grind them. She’d grind them in a *wira* dish, after chopping them up well. It’s to rub into aches and pains. You know, the kernel inside. The skin on the outside they’d eat as food. And they would rub the kernels into the back. If you had a backache, you’d rub it in. Sleep the night, then get up and think: ‘Hey! That pain of mine has gone!’ With the aid of that medicine, I’ve recovered. They’d just get better and go off for game. Yes, that’s all I’m saying—Mollie Everard [23: 37–7].

## Quandongs, their distribution and use

**Fig 2:**
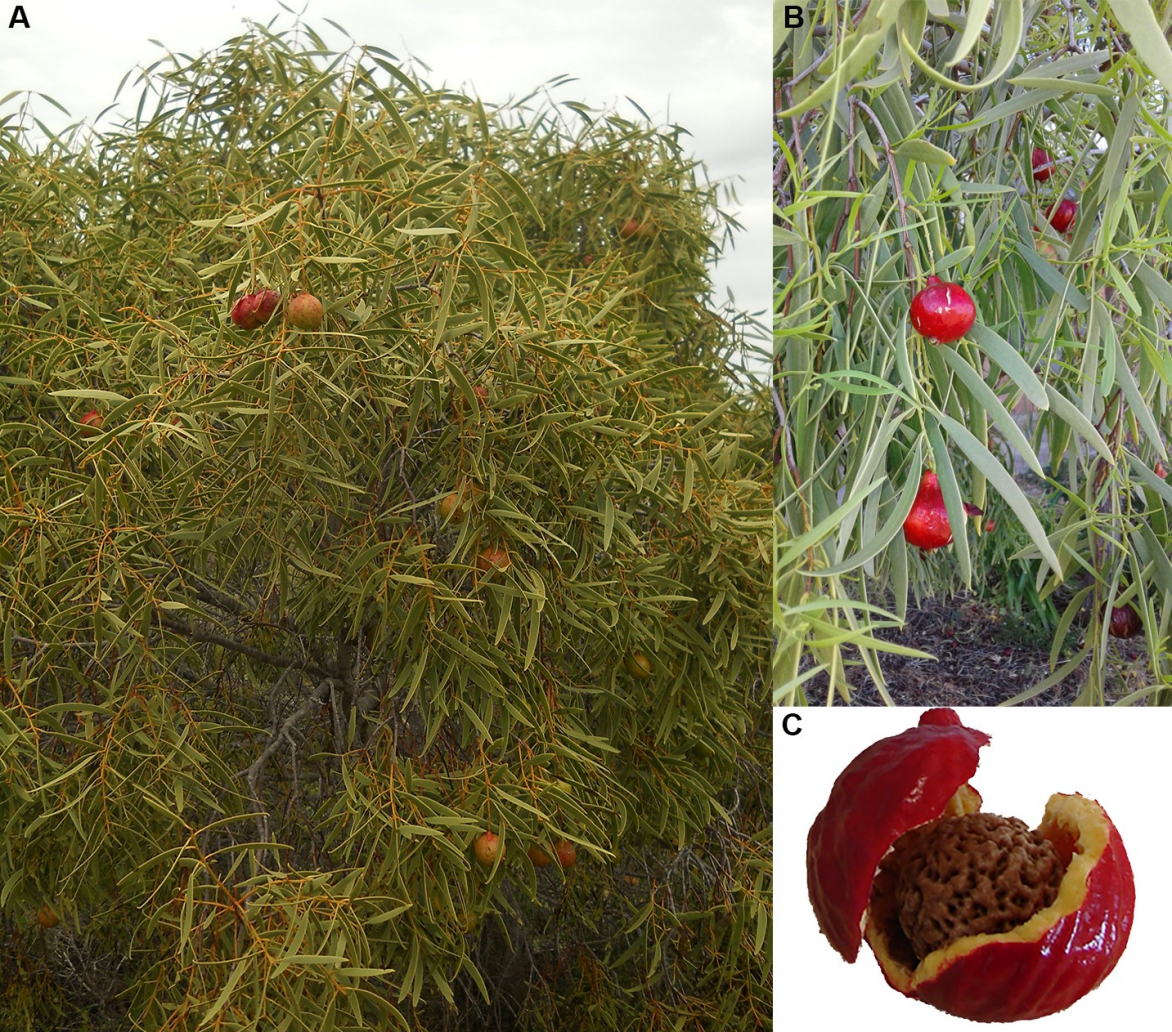
Quandong tree and fruit. Quandong trees are small [<6m] with bright red fruit. Source: Atlas of Living Australia website. Accessed Nov 2018-11-21, https://www.ala.org.au/, ©OpenStreetMap contributors]. Photographs by Susan Wiser [left], David Muirhead [top right], Loxley Fedec [lower right]. This material is released under the Creative Commons Attribution 3.0 Australia Licence ‐ http://creativecommons.org/licenses/by/3.0/au/].

The desert quandong, native peach or bush peach, *Santalum acuminatum* (R.Br.), is a favourite Aboriginal food found throughout the arid and semi-arid zones of southern Australia extending into the centre of the continent [24,25]. Like most plants of the sandalwood (Santalaceae) family, it is hemiparasitic, relying in its early stages on a range of host plants for water and soil nutrients but not for sugars. It is a small shade-giving tree that varies in height from one to six metres (Fig 2). Flowers appear in summer forming fruit that ripen the following spring and summer [26,27].

Depending on the season, trees are laden with bright red mature fruits that vary in size from 15–25 mm diameter with flesh 3–5 mm thick. The fruit is dry-textured and tart-tasting but the sugar content increases when they are dried. Like many wild fruits, quandongs are rich in polyphenols, have a greater antioxidant capacity than the blueberry, and are efficient inhibitors of pancreatic lipase [28,29]. The fruit is rich in Vitamin E components, magnesium, zinc, selenium and iron [29,30]. The central stone contains a large single kernel that is rich in oils and protein [31]. The bark and leaves also have anti-microbial properties [32,33]. While individual trees are common, particularly in sandy or rocky country, quandongs are not evenly distributed across southern Australia but cluster in groves throughout their range (Fig 1). Quandongs and their use by Aboriginal groups have been documented in museum collections and historical records since first European settlement in arid and semi-arid areas across the continent, including throughout the MDB (the focus of our research here).

There are several well-known centres in the MDB, including West Wyalong on the Lachlan River catchment, central NSW. The name *quandong* is the anglicised version of the Wiradjuri word *guwandhang*, although according to Wiradjuri Aboriginal people in West Wyalong, a town within our study area, *wyalong* is the local Indigenous word for a hard-shelled nut or quandong. They grow in clusters on the edge of forests, in patches of open scrub, and in groves on sandy rises. When Thomas Mitchell travelled along the Murray River in 1838, he noted that wherever he camped on raised, dry ground, he found abundant groves of quandongs and anticipated their commercial use [34]. When Charles Sturt travelled up the Murray River in 1844, *Nadbuck*, one of his Aboriginal guides collected a large quantity of quandongs for the party. Sturt later regretted that they had not saved more to ward off the scurvy that affected them further on in the trip [35]. Quandong nuts were also found cached in a large rock shelter at Puritjarra, Central Australia [36].

Dreaming stories often reference a symbiotic relationship between quandongs and emus. The birds eat the fruit in large quantities, swallowing the nuts. Their stone-filled crops begin the digestive process so that as they wander the landscape they excrete a ready supply of quandong nuts whose germination is assisted by fertiliser [37]. Aboriginal people recognised the role of the emu in aiding their distribution.

The location of quandong groves was well known to local Aboriginal groups who harvested the fruit in quantities for short or long-term use. The fruit can be eaten raw but dried fruit was particularly valued because of its sweetness and its ability to be preserved and readily transported [21,22,38]. Dried fruit was peeled from fallen quandongs and excess fruit dried, pounded and rolled into balls or cakes for later use [24]. This dried fruit could then be reconstituted with water into a paste that formed an important food source in times of scarcity: ‘It’s really good when you don’t have meat’—Pompey Everard [23: 35].

Inside the fruit is a single, large round nut with a very hard casing; this contains a large edible kernel about the size of a pea which was highly prized as food, raw or roasted, or was ground into an oily paste or salve valued for its capacity to ease rheumatism and aching joints [23: 32–7] or to relieve tooth ache and gum boils (Beryl Carmichael of Menindee, personal communication). The crushed kernels were also used among Barkandji women of the Darling River as skin cream and hair conditioner (Dayle Doyle and Dot Stephens of Menindee, personal communication).

The nut is very hard to crack and hitting it with a hammer risks pulverising and mixing the kernel with nut fragments. This is not a trivial concern in Aboriginal Australia. Teeth are an extremely valuable possession that must last a lifetime. Although their considerable wear, with ensuing abscesses and tooth loss, is well documented, the prevalence of tooth fractures (as might be expected when chewing food with large, hard shards) is low [39]. Given the high value of the kernels for food, medicinal and cosmetic purposes, extracting the kernels warranted specialised implements located in productive areas in order to capitalise on the seasonality and distribution of the fruit.

## A study of ground stone in the murray darling basin

**Fig 3:**
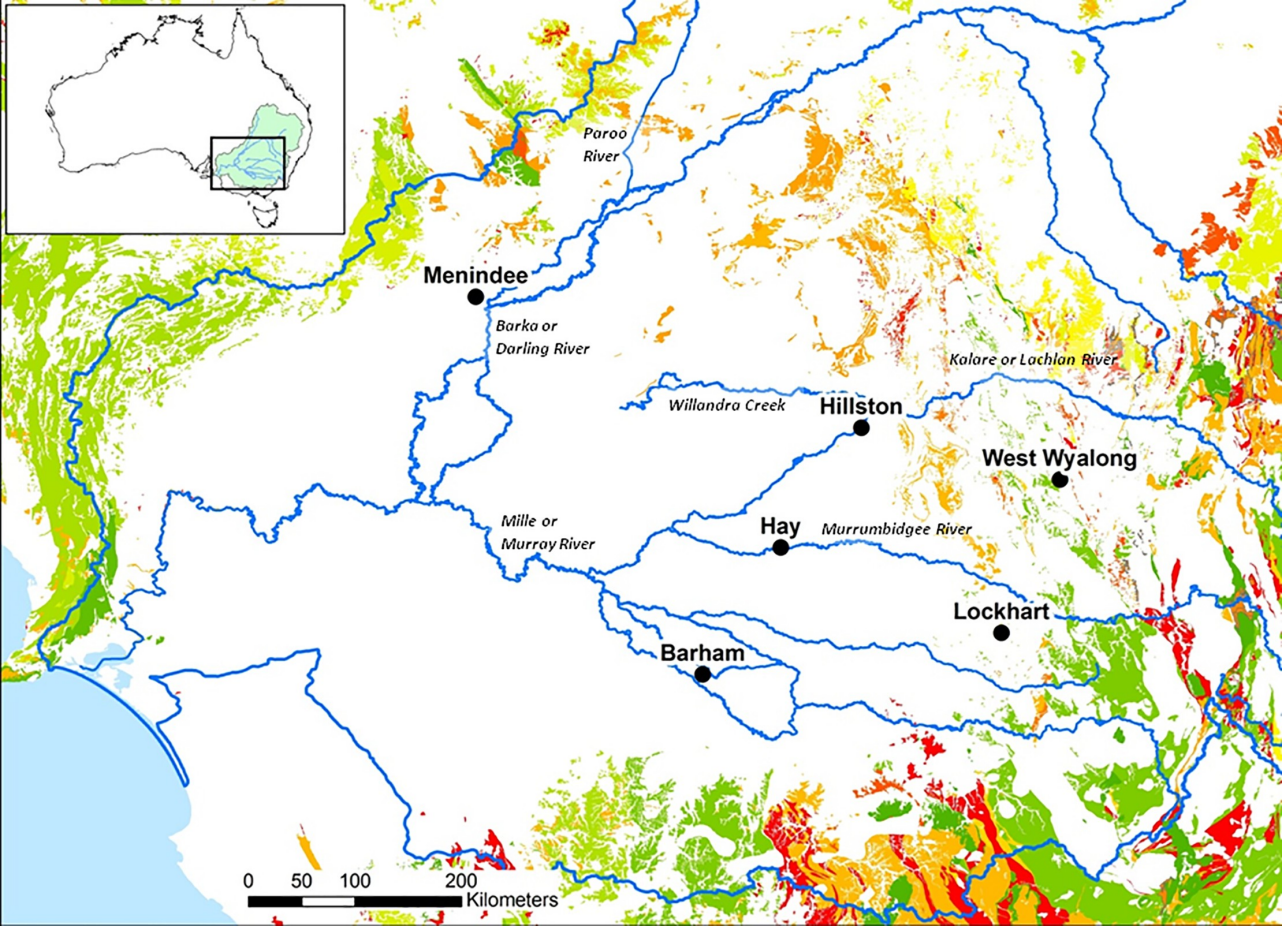
The study area with relevant geology and collection locations. The southern part of the Murray Darling Basin, Australia, with major river systems, locations in the current study and outcropping quartzites and sandstones. (Map created by CP using data from Geoscience Australia. ©Commonwealth of Australia (Geoscience Australia) 2012. This material is released under the Creative Commons Attribution 3.0 Australia Licence ‐ http://creativecommons.org/licenses/by/3.0/au/).

This paper forms part of a large-scale study of the distribution of ground stone implements and trade in the southern MDB. The study area, measuring roughly 550 km (east-west) x 500 km, spans five river systems: the Murray River to the south, and the Murrumbidgee, Lachlan, Darling and Willandra Rivers heading north towards the Queensland border (Fig 3).

The research represents a collaboration between Aboriginal groups, rural museums, farmers and other property owners. So far 1,327 grinding implements have been examined from 12 collections held in Barapa Barapa, Nari Nari, Wiradjuri, Ngiyampaa, Barkandji and Gunu traditional territories. Some collections are held by Local Aboriginal Land Councils or other Aboriginal groups. Some are located in rural museums. Others have been collected over several generations on farm properties in the course of agricultural and pastoral activities or exposed by erosion. Property owners have collaborated with local Aboriginal groups to ensure that collections are catalogued, studied, and information provided to the local community. Sometimes the collections have formed the basis of interpretive displays at local centres such as the Flywheelers Museum at Barham or the West Wyalong Local Aboriginal Land Council. Often, though, Aboriginal groups have asked that the collections remain on country, close to where their ancestors used and left their axes, grinding dishes and other valued evidence of past activities.

For full details of location of collections, including geographic coordinates, repository information, item numbers, and accessibility see S1 Table. No permits under the National Parks and Wildlife Act 1974 (New South Wales) were required for the described study. All items examined had been collected prior to the enactment of the legislation. No specific permissions from regulatory authorities were required for this study but all collections were examined only with the permission and approval of the relevant Aboriginal custodians and collection holders. These are identified in S1 Table.

In a study of trade and distribution, sample areas will inevitably be large. Given the paucity of stone sources throughout the Basin (Fig 3), virtually all implements represent trade activity into local Aboriginal territories from elsewhere. Sometimes land owners have been able to identify the original location of a recovered item but the majority can be provenanced only to the property level. In a large-scale study such as this, though, the location of an item within 20 km^2^ is an appropriate sampling strategy in an area of 188,000 km^2^.

Biases in the collections studied are to be expected. Collection might have been influenced by size with larger objects, for instance, being more visible. While visibility is a major factor in archaeological fieldwork, in this part of the country where there is virtually no locally outcropping stone and a long history of drought and wind-blown erosion denuding the ground, every item is noticeable and many collections contain small items. Certain types of objects may have been selected preferentially: rare items, unfamiliar stone material, particularly beautiful pieces, for example. Generally, though, the collections studied appear representative of their areas, reflecting variation in local ecologies, such as an increase in soft seed grinding implements in those areas where summer grasses predominate.

Within the MDB, grinding, pounding and chopping implements dominate collections in the form of soft seed grinding dishes and top stones, mortars and pestles, hammers and axes—all items that are usually included under the broad category ‘ground stone implements’ [10,15,40]. Material in the collections ranges from soft sandstone through to the hardest quartzite and igneous rock. Clearly, grinding and pounding pieces appeal to the collectors and are highly valued by Aboriginal people. One of the attractions of these objects is the visible wear that is the result of ancestral elbow grease. The deep hollows in grinding dishes, the polish, cracks or pits in mortars, all provide tangible links down through the generations. Many of the wear facets can only be the result of continued use measured in centuries, not years or days.

## Preliminary description of the collections: Typology and taxonomy

A stumbling block in the classification of many grinding and pounding implements has been their multi-functional nature where the same tool may bear evidence of a range of tasks and actions (or modes of use). For example, Caroline Hamon [13] identified use-wear on *broyons* that were used interchangeably as crushers and grinders, though not for cracking nutshells [13]. Actions include abrading or sharpening; grinding or husking; mincing, crushing, pounding or hammering; chopping, splitting, sharpening or cracking [41,42]. Multi-functional tools are particularly evident in regions such as the MDB, where stone of any kind is a scarce resource traded from surrounding areas, and where variation and overlap in form, function and raw materials is evident [43]. Form and function can also change over time as implements break or are worn down. A grinding dish becomes a top stone or whetstone; a broken mortar is recycled as an anvil or hammer. Long-term use is a common feature as evidenced by the depth of grinding facets, size reduction and recycling. When a single item is clearly used for a range of different functions, type categories cannot be mutually exclusive and are based on arbitrary criteria. One of the aims of this study has been to see if there is any clear distinction between multi-purpose and single purpose implements that might help us to distinguish between generalised and specialised activities, and evaluate their contribution to foraging strategies.

All 1,327 stone implements within the MDB collections have been catalogued, measured, weighed and described. While recognising that typologies do not work well with these assemblages, given the number of multi-purpose implements, items have been allocated within a convenient typology that draws largely on Frederick McCarthy’s classifications [10]. A specialised item, the portable mortar and pestle (PMP), was added.

The PMP is a small item, usually a rounded rectangular block of quartzite with small mortar facets on one or both large faces, pestling marks around the margins and often hammer damage. It is designed to be used with another piece [often glossed as ‘big and little brothers’ in local languages]. This can be another PMP or a kulki, a small round piece (<400 g) usually with a pit in either flat face, surrounded by a small grinding facet. The margin is usually a continuous pestle facet, to be used with the PMP. Hammers grade into PMPs, although they are often rounder in cross section, with defined hammer damage on either end, consisting of crushed and fractured quartz grains and occasional ring cracks. Axes should properly be termed hatchet heads, but common usage will be followed here. A second new category, the blocksplitter, describes axes that are much thicker and heavier than usual. These may be used as wedges to remove slabs of bark or wood from trees.

The current study has also applied a taxonomic approach where items have been identified on the basis of empirically observable characteristics that cross-cut typologies and permit alternative groupings. Functional attributes include grinding facets, pits, mortar and pestle facets, hammer damage, ring cracks and surface texture. These attributes identify particular functions that can then be quantified across the range of classificatory types. Pits are distinguished from percussion or pecking marks. The former are larger holes that are the result of continued hammering, whether of stone, nut or wood. The latter are the result of a single hit of stone on stone. Ring cracks may develop around the point of impact without any quartz grains being dislodged.

As the collections were examined, a provisional category ‘quandong stones’ was proposed to describe a number of distinctive implements. Although only 24 in number, comprising only 2% of the total collections, they are visibly different from the other large implements and bear a similarity to North American nutting stones [8]. On seeing two in a collection, Robert Clegg, Wiradjuri Aboriginal elder, immediately identified them as ‘nutcrackers’ (personal communication). Quandong stones are described in the following section. To further evaluate them as a distinct category, their macroscopic features are then compared with those of other anvil stones and the occurrence of pits is examined across all implement categories in the collections. We analyse depth and shape of pits and the weights of implements with pits. As a pilot study, we report on use-wear and residue analysis of four available quandong stones, a pestle, and a mortar all from the same area.

## Quandong stones: A provisional new category

### Description of macroscopic features

**Fig 4:**
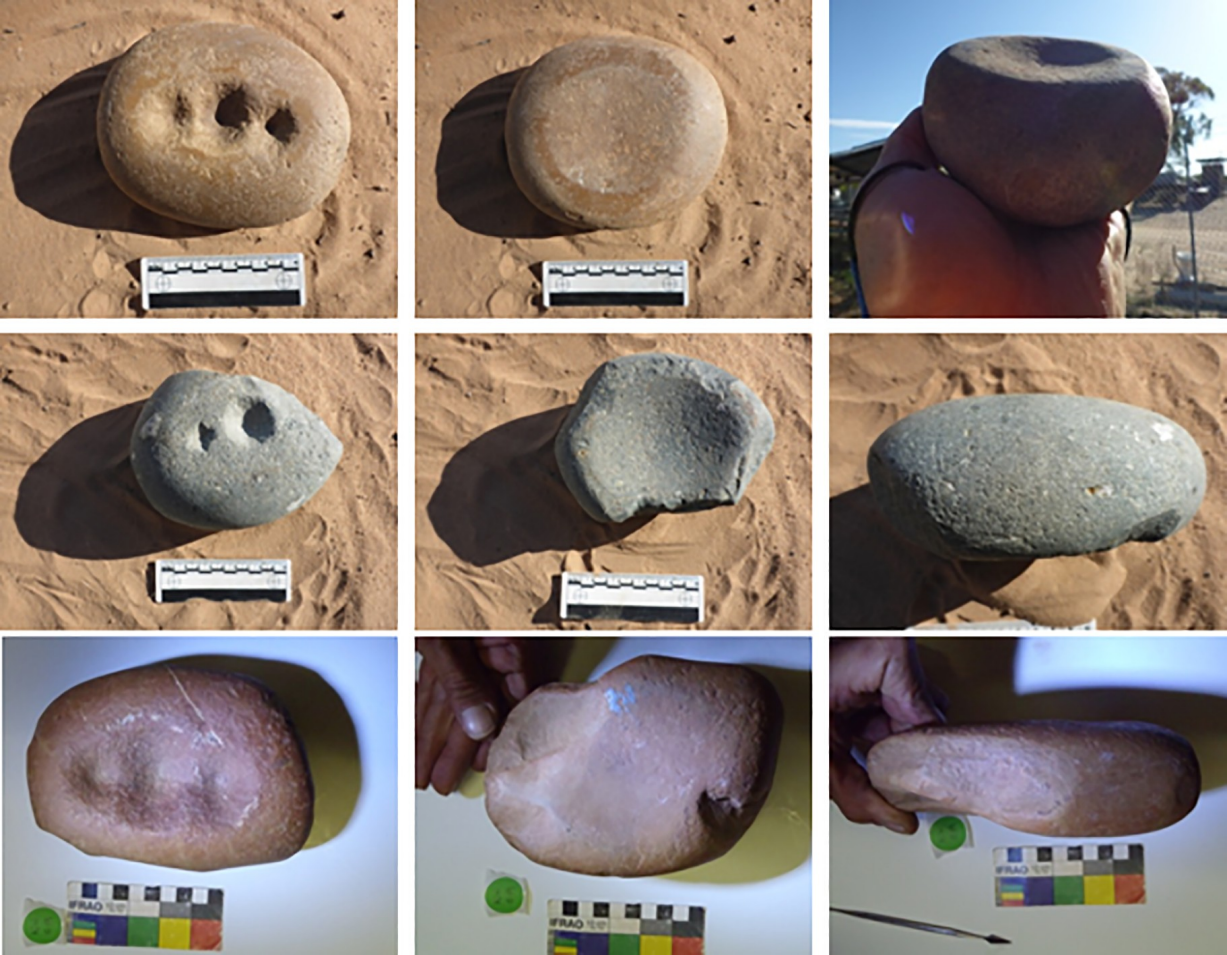
Classic forms of quandong stones. The classic form of quandong stone is a flattish quartzite cobble with multiple large pits on one face [left], a small mortar bowl facet on the other [centre], and a flat faceted surface surrounding the mortar bowl facet [upper right]. The naturally smooth surfaces of these water rolled cobbles have been polished through handling and use [centre right]. The two upper are Barkandji group, Kinchega National Park, Darling River, KNP-01, KNP-02; the lower is Wiradjuri group, West Wyalong LALC WW019). Photographs by CP.

**Fig 5:**
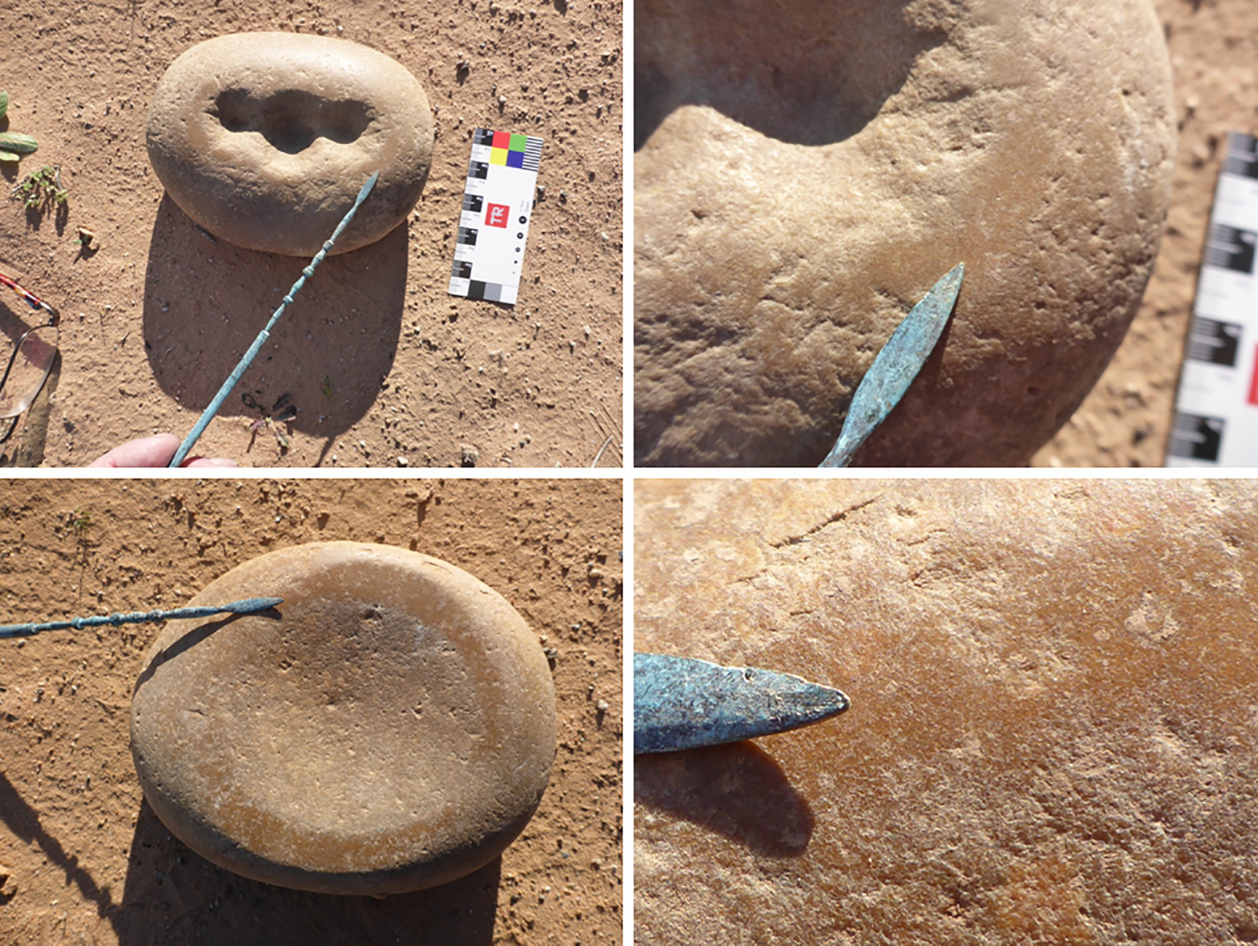
Detail of surfaces of quandong stone KNP-01. The classic Quandong Stone has more than one pit on the upper surface and a well-defined mortar bowl on the lower. Note the distinct polished areas on both surfaces. [Kinchega National Park, Darling River, KNP-01]. Photographs by CP.

The objects we have come to call ‘quandong stones’ were first recognised as flattened, oval cobbles weighing around 1.5 kg (Table 1; S1 Table). The cobbles are hard, tough, brown quartzite, with a smooth, stream-rolled surface. The ‘classic’ type is characterised by one or more quite deep circular pits either in the centre of the cobble or arranged in a row along the long axis (Fig 4; Fig 5). On the obverse face, a mortar bowl is surrounded by a polished facet.

**Table 1:** Summary statistics for quandong stones.

Variable	mean	minimum	maximum	SD	n
Length (mm)	149.0	107	204	29.8	24
Width (mm)	119.6	63	187	31.9	24
Thickness (mm)	57.5	35	115	17.4	24
Weight (g)	1608.6	543	3226	760.0	22
√(thickness/weight)	0.20	0.13	0.29	0.04	22
√(length x width)	133.0	87	195	29.7	24
Pit 1 depth	8.1	2	15	3.7	14
Pit 2 depth	7.8	2	17	4.6	13
No. of grinding facets	1.3	0	3	0.8	24

SD: standard deviation, n: number of observations.

Distinct polished areas are visible on both faces of the quandong stones. This is not the normal smooth surface of water-rolled cobbles, but a sheen imparted from considerable handling and use. The even distribution of sheen on both faces argues against natural taphonomic processes and is not consistent with sand blasting or desert varnish. In addition, distinct highly polished facets are equally visible on both faces of many quandong stones. On the side with pits, polished areas are more pronounced around the rim of each pit; on the mortar side, the most developed polish zones occur on the worn down rims of the depressions. While the interior of the symmetrical pits did not appear polished, the sides were even and smooth with no visible evidence of percussion fractures.

The pits are approximately 8 mm deep and 30 mm across. Given the depth and even, symmetrical shape of these pits, their use for cracking quandongs was an obvious hypothesis. Visual inspection of the pits shows smooth and rounded quartz grains on the walls and base, and a rounded lip on the surface, not consistent with stone on stone percussion. In addition, many of the pits are too deep and steep-sided to allow finger and thumb to hold a quartz pebble for fracturing.

**Fig 6:**
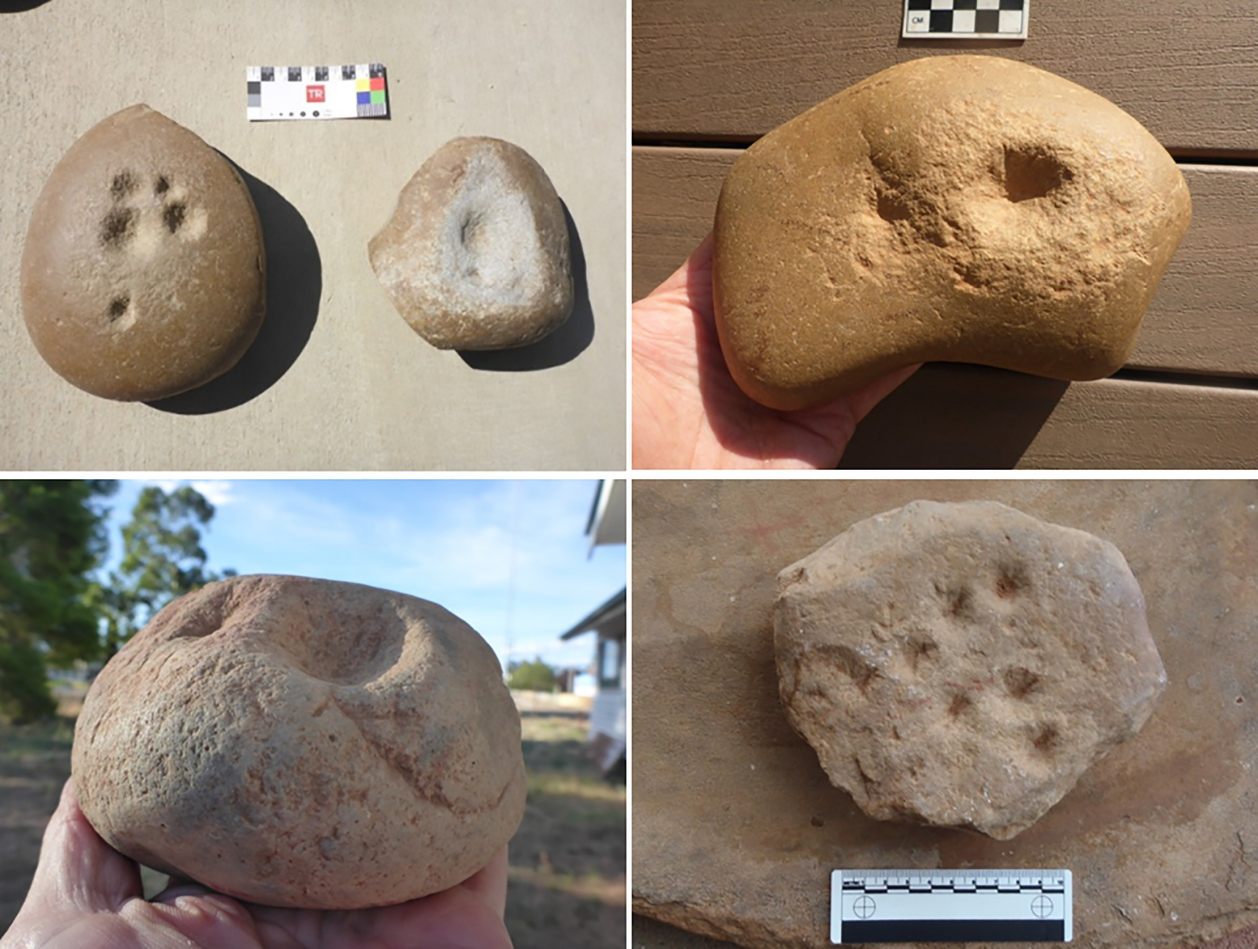
Variety of quandong stone forms. The variety of items that came to be classified as quandong stones maintains several features of tough material [quartzite], deep and smooth pits, polish and larger size. Top left: Hay, Murrumbidgee River, H18-08 and H18-09; Top right, Lockhart, Murrumbidgee River catchment, OUR-02; Bottom left, Hillston, Lachlan River; HHS-B02, Bottom right, Hillston, MCG-01, Lachlan River. Photographs by CP.

Once identified as a type, morphological variation became apparent. While most had one or more pits on one face and a mortar on the other, some mortars had a pit in the centre of the facet; in some, pits were in different stages of development; a few were made of quartzite blocks, not river cobbles, but all fell largely within the parameters identified above (Fig 6; S1 Table).

**Fig 7:**
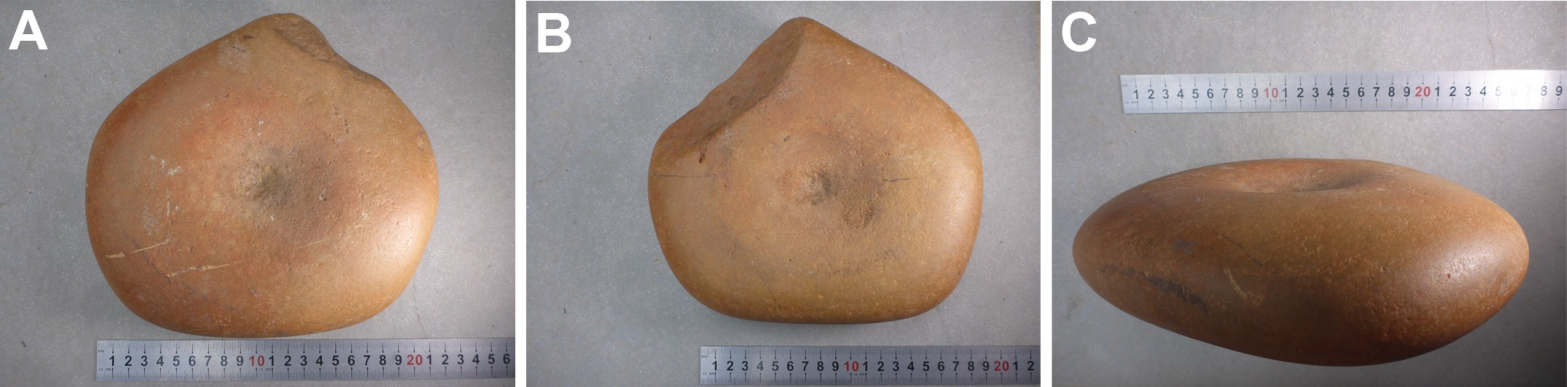
Quandong stone WW050A. Specimen WW050A collected from a quandong grove under a tree. This quandong stone has a mortar facet on each face with a pit in the centre of each. Photographs by CP.

One item (WW050A; Fig 7) had been collected from a quandong grove under a tree. This had mortar facets on both faces with a pit in the centre of each. A pestle (WW050B) had been found alongside it.

**Fig 8:**
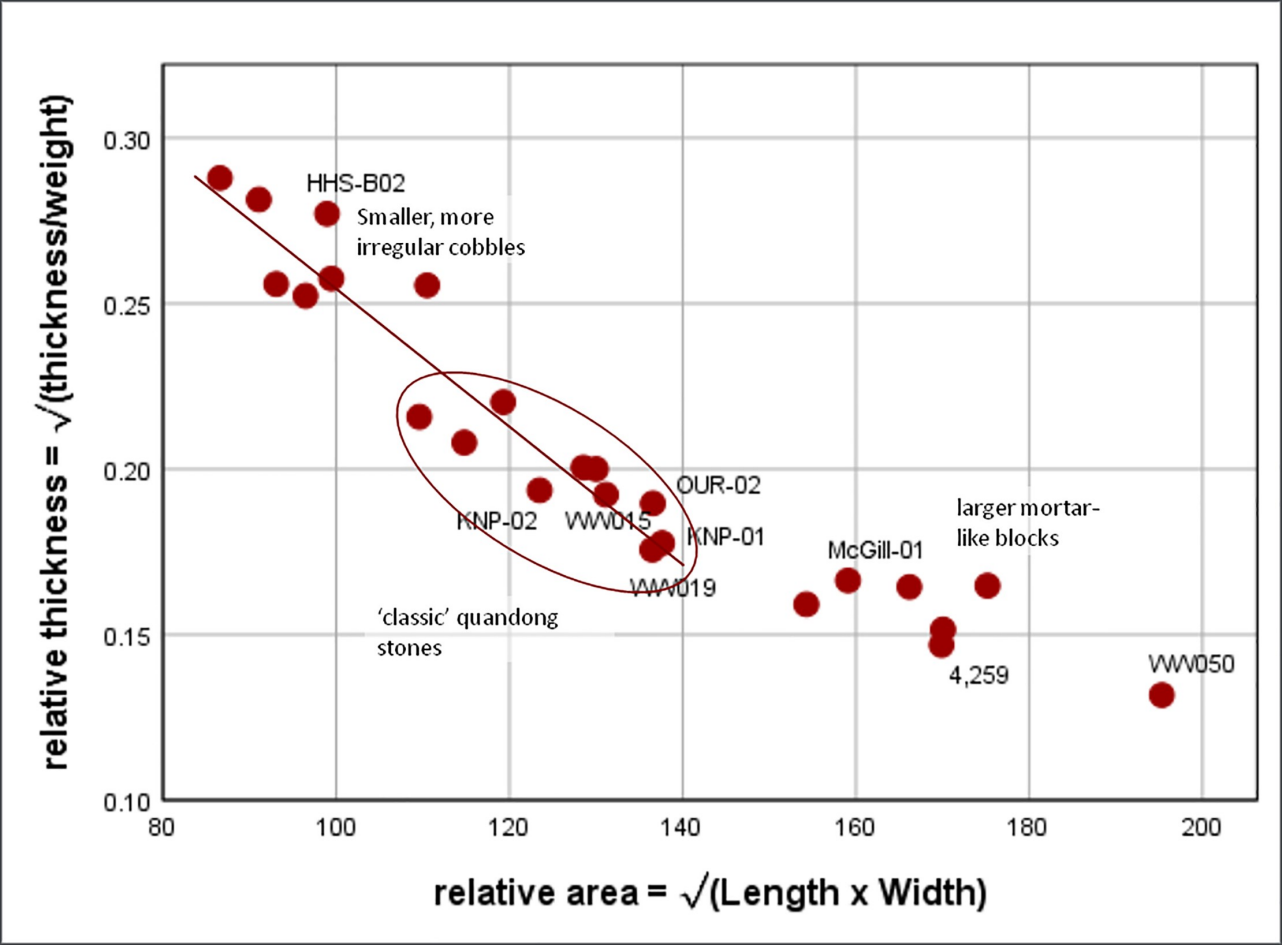
Relative thickness of quandong stones. Relative thickness ($\sqrt{\left(thickness/weight\right)}$) plotted against relative area ($\sqrt{\left(length\;x\;width\right)}$). Perceived forms are clearly differentiated, with the ‘classic’ quandong stones circled. The smaller, more rounded items are relatively thicker and cluster in the upper left. The larger, more typically mortar-like items are relatively thinner and cluster on the right. The regression line is calculated only for the items on the left, illustrating their relative similarity contrasted with the mortar-like items on the right. Labelled items are illustrated in other figures.

The variety of size and shape among items is partly a result of their different source materials. Some of the smaller items are amorphous quartzite cobbles. The larger tend to come from slabs and could be classified as mortars. The classic items are in-between and tend to be river cobbles. Relative thickness ($\sqrt{\left(thickness/weight\right)}$) plotted against relative area ($\sqrt{\left(length\;x\;width\right)}$) provides some further clarification of the variety (Fig 8). Although such a plot produces an auto-correlation, given that weight is more or less completely determined by length, width and thickness, the clustering along a linear trend clearly differentiates and is indicative of the overlap among the multi-purpose items. Relative thickness of quandong stones is partly a function of the size of the object, but is much greater than sandstone dishes, whose values are typically <0.10.

### Analysis of pits across implements in the collections

Quandong stones clearly function as a base or anvil and need a stone hammer or some form of wooden mallet to complement them [5,13,40,41]. Pits would also be visible in this percussion instrument [44]. Grinding the kernels on the second face would also require a pestle. Only one potential pair (WW050A and pestle WW050B) was seen, in the collections, but many items could serve the purpose.

McCarthy’s classification of stone implements includes a category ‘anvil stones’ that have ‘one or more pits on the upper and lower surfaces and sometimes on the sides and ends’. He includes in this category a type ‘common in Victoria’, that he describes as a large cobble ‘bearing numerous pits on one surface’ [10: 55]. It is possible that these were quandong stones.

A pit starts with percussion marks in an implement that develop into a shallow indentation and then deepen into a steep-sided hole. Pits across all implements in the present study vary in depth from 1–2 mm to 20 mm and are typically 20–30 mm in diameter with a rounded lip at the surface. They may lie within a mortar bowl facet or on the side of an axe. They form part of the definition of the kulki [10: 55], a portable multi-purpose mortar, pestle, hammer, nut cracker. The term ‘anvil pit’, however, has come to be associated with flaked stone tool work, particularly bipolar and microlith flaking. Such a function makes sense, particularly in areas of abundant quartz pebbles, where an anvil platform may be necessary on account of the small size of the piece being flaked [11]. Anvils have also been associated with a range of other functions including breaking up woody seeds and nuts [44], smashing animal bones to obtain marrow, opening shellfish, or mixing pigments [41]. They functioned as a generalised kitchen implement. Other suggestions for pit function have included ‘providing a grip for the finger and thumb when in use’ [45: 73,46: 385].

As a consequence, cobbles with pits, and pitted stones more generally, have been identified as a controversial, confusing tool type [3]. Recognising this, the following analysis moves beyond typological classification to analyse the distribution of anvil pits as a functional attribute across all implements in the MDB collections.

#### Frequency of pits across implements

Pits in stone implements in the MDB collections are fairly common—18.6% (n = 1,327) of all implements in the current study. From a functional perspective, pits are an attribute of several different types of implement (axes, mortars and pestles, hammers and quandong stones) and contribute to understanding the multi-purpose nature of implements throughout the region.

The 1,327 items in the MDB collections can be broadly divided into ground stone implements made of soft sandstone, indurated sandstone, quartzite, and igneous rock. Pits do not occur in sandstone that risks breaking if used as an anvil; they are only found in quartzite and igneous stone of greater strength, hardness and toughness. Of the total study sample, 694 or 52% are sandstone implements, including types such as soft-seed grinding dishes, top stones and whetstones. None of these implements contained a pit and are therefore excluded from this study. Pits were documented in the remaining implements (632 = 48% of the total). Pits were found across all types of hard stone implements (Table 2). A total of 33% of these hard stone implements had at least one pit; 67% had no pits (Table 2). A total of 24 (~2% of 1,327 items in the MDB collections) are classified as quandong stones (S1 Table).

**Table 2:** The frequency of pits for each type of hard stone implement.

Hard stone implement type	N	pits absent %	pits present %	% implements with n pits							
				n = 1 %	n = 2 %	n = 3 %	n = 4 %	n = 5 %	n = 9 %	n = 15 %	Total %
Axe	184	**85**	**15**	**10**	**4**	**0**	**1**	**0**	**0**	**0**	100
Blocksplitter	6	**50**	**50**	**17**	**33**	**0**	**0**	**0**	**0**	**0**	100
Mortar	172	**74**	**26**	**19**	**7**	**0**	**0**	**0**	**0**	**0**	100
Pestle	72	**92**	**8**	**8**	**0**	**0**	**0**	**0**	**0**	**0**	100
PMP	48	**42**	**58**	**35**	**21**	**2**	**0**	**0**	**0**	**0**	100
Hammer	85	**44**	**56**	**27**	**24**	**4**	**1**	**1**	**0**	**0**	100
Kulki	41	**27**	**73**	**37**	**27**	**7**	**2**	**0**	**0**	**0**	100
Quandong stone	24	**0**	**100**	**20**	**48**	**20**	**4**	**0**	**4**	**4**	100
totals	632	**67**	**33**	**18**	**12**	**2**	**1**	**0**	**0**	**0**	100

‘pits present %’ is the sum of ‘% implements with n pits’.

Pits are common among the axes, with a preference for pits in thicker axe bodies (listed as blocksplitters)—a sensible decision on such an expensive item. About one quarter (26%) of mortars have pits, while most PMPs (58%) and kulki (73%) have pits. All quandong stones (100%) had at least one pit, but that was a defining criterion.

A distinction between tool types is also apparent in distributions of the number of pits per item. Multiple pits are common among quandong stones (80%), rare in the large mortars (7%), non-existent in pestles (0%) but by no means uncommon among the portable multi-purpose items: portable mortar/pestle (PMP 23%), hammer (30%) and kulki (36%).

#### Depth and shape of pits

**Fig 9:**
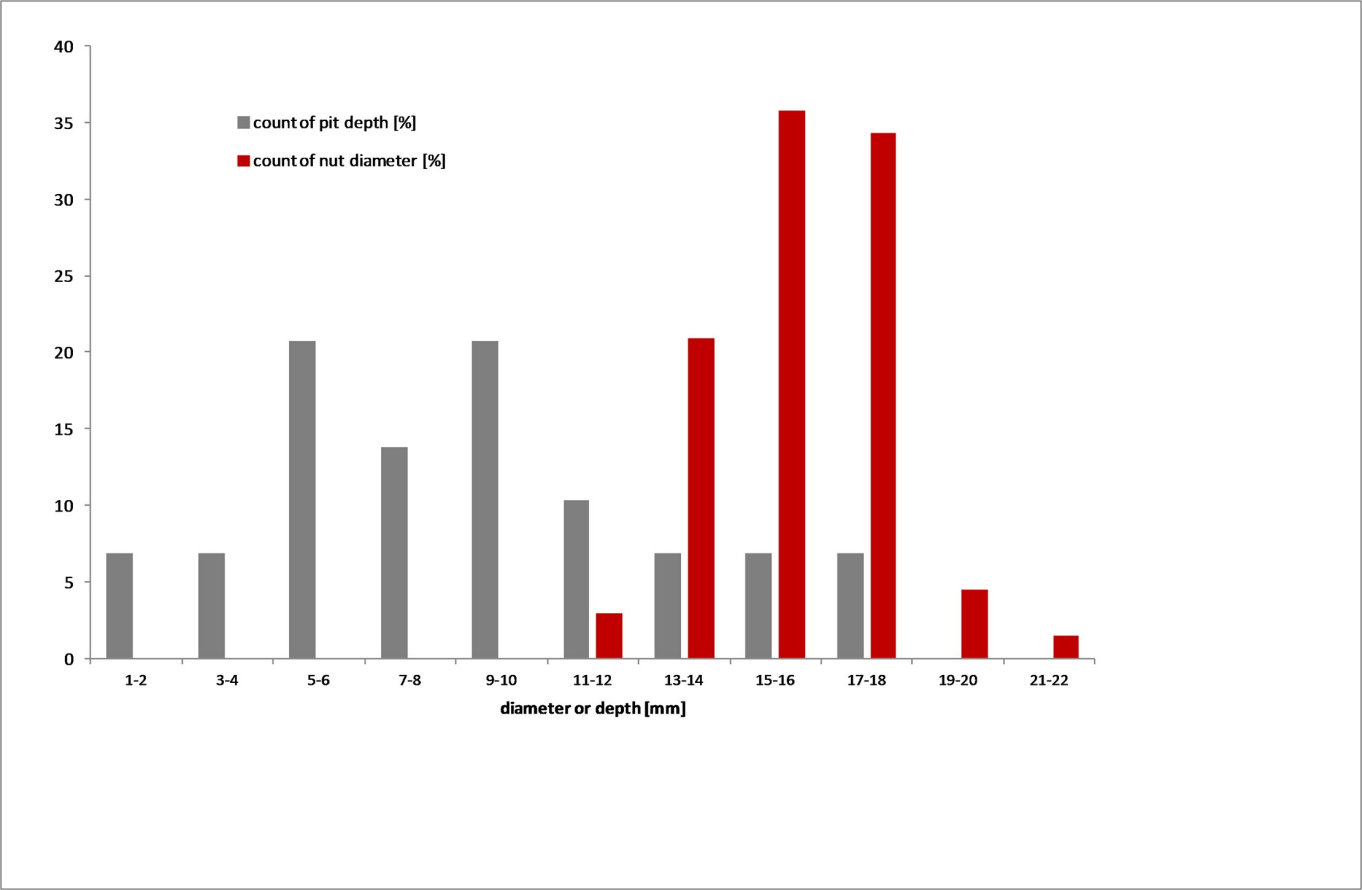
Pit depth and quandong nut diameter. The distribution of nut diameter is normally distributed, as expected; while that of quandong stone pit depth varies more widely. This might be the result of the sample having younger and more developed pits, as well as differentiation by function, with an excess of deeper pits for nut cracking.

The depth of pits is relevant to differentiating tool types and an assessment of their potential use (Fig 9). While we might expect to find a pit in an object at any stage in its lifespan, the range, mean and maximum depths may be indicative of functional specificity. Among the axes, pits are shallow, with a mean of 2.6 mm. Portable implements also have shallow pits with means between 3.2 and 3.7 mm; the larger mortars average 7.0 mm depth and quandong stone pits are deepest at 8.7 mm.

Quandong nuts must sit proud of a pit in order to be cracked efficiently. The nut averages 16.4 mm in diameter, with shell thickness 2.2–2.4 mm. In order to protect the kernel from smashing, an ideal differential would be on the order of 3–6 mm. Quandong nuts do not vary much in diameter (Fig 9), and are typically greater than the depth of a quandong stone pit, particularly if one accounts for shallower pits being ‘younger’. The differential in maximum depth of pit and nut diameter likely indicate a functional maximum to the pit.

**Fig 10:**
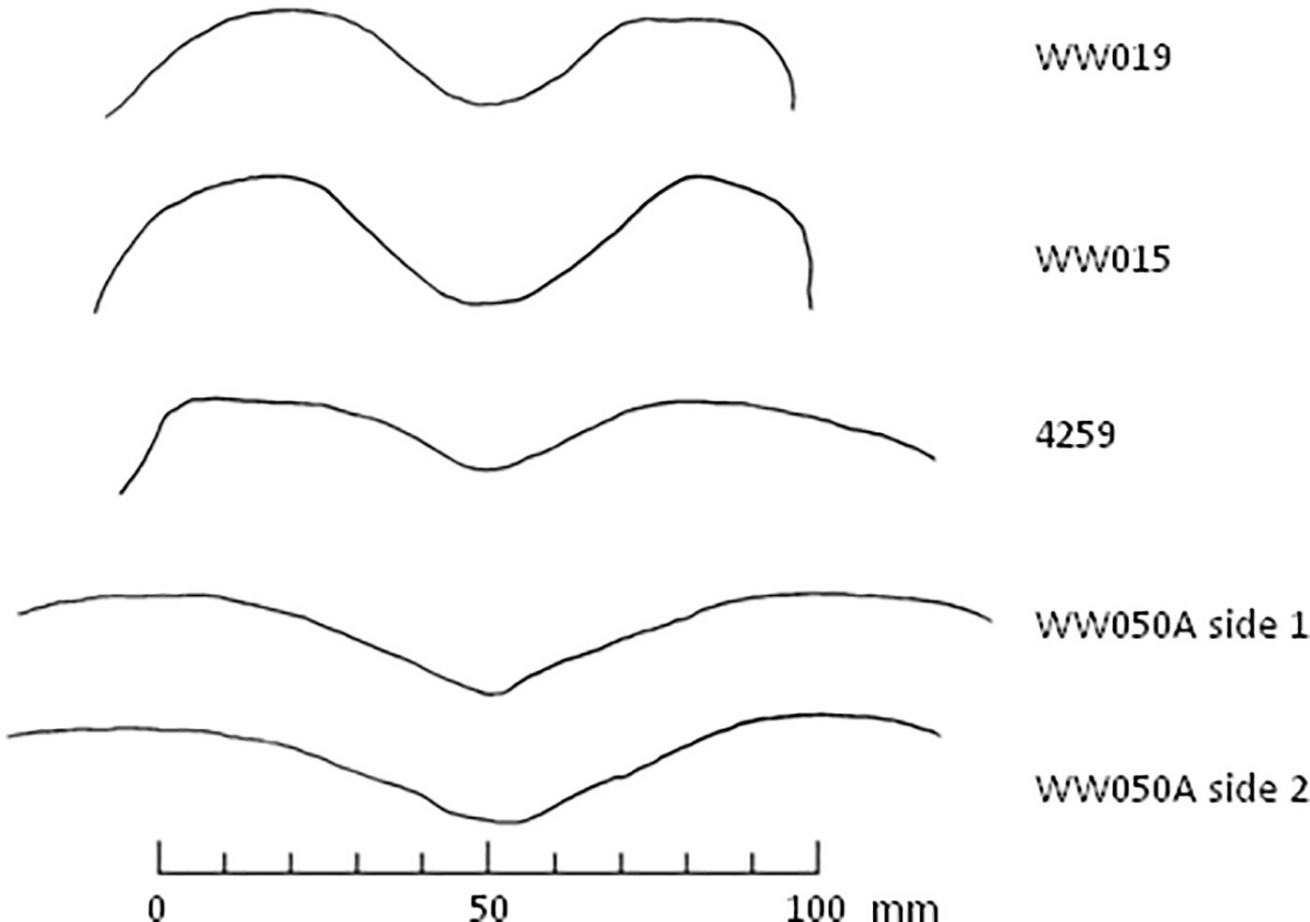
Cross section drawings of five quandong pits. The upper two are from ‘classic’ forms; the lower three are pits within mortar bowl facets.

The shape of quandong stone pits is also distinctive. They are consistently circular, rounded at their base, and symmetrical in profile. They have a smooth finish unlike many pits on other items which may be irregular and often show quartz grain fracturing. Many pits, particularly on classic forms, are surrounded by a flat rim that is typically smooth and polished. Symmetry is evident in the cross-section drawings of the quandong stones selected for use-wear analysis (Fig 10).

#### Weight of all items with pits

Almost all stone found in the MDB has been imported from elsewhere, whether sandstone slabs for grinding, harder quartzite blocks for mortars, igneous rock for axes, or river cobbles used for a range of functions. Weight is a significant factor in daily life, and portability based on weight can give insights to foraging strategies. Grinding dishes, mortars and probably quandong stones are too heavy to transport on a daily basis and would be cached in particular locations ready for use. Their portable counterparts: hammers, PMPs, kulki and axes weigh around 0.5 kg (Table 3). It was common for women to carry pounding stones of some form in their bags for smashing roots, opening mussels and a range of other activities in their daily food quest [47,48,49: 141,50]. Large items are found either at residential sites near reliable water or distributed over areas where seasonal resources require processing in situ [20,43].

**Table 3:** Average weight of items with pits.

Object type	mean (g)	SD	n
Mortar	2,361	3,141	73
PMP	663	271	35
Kulki	389	237	39
Quandong Stone	1,532	669	24
Axe	504	341	150
Blocksplitter	849	399	7

The large standard deviation (SD) of mortars is the result of a wide range that includes several exceptionally large items documented from locations along the Darling River.

**Fig 11:**
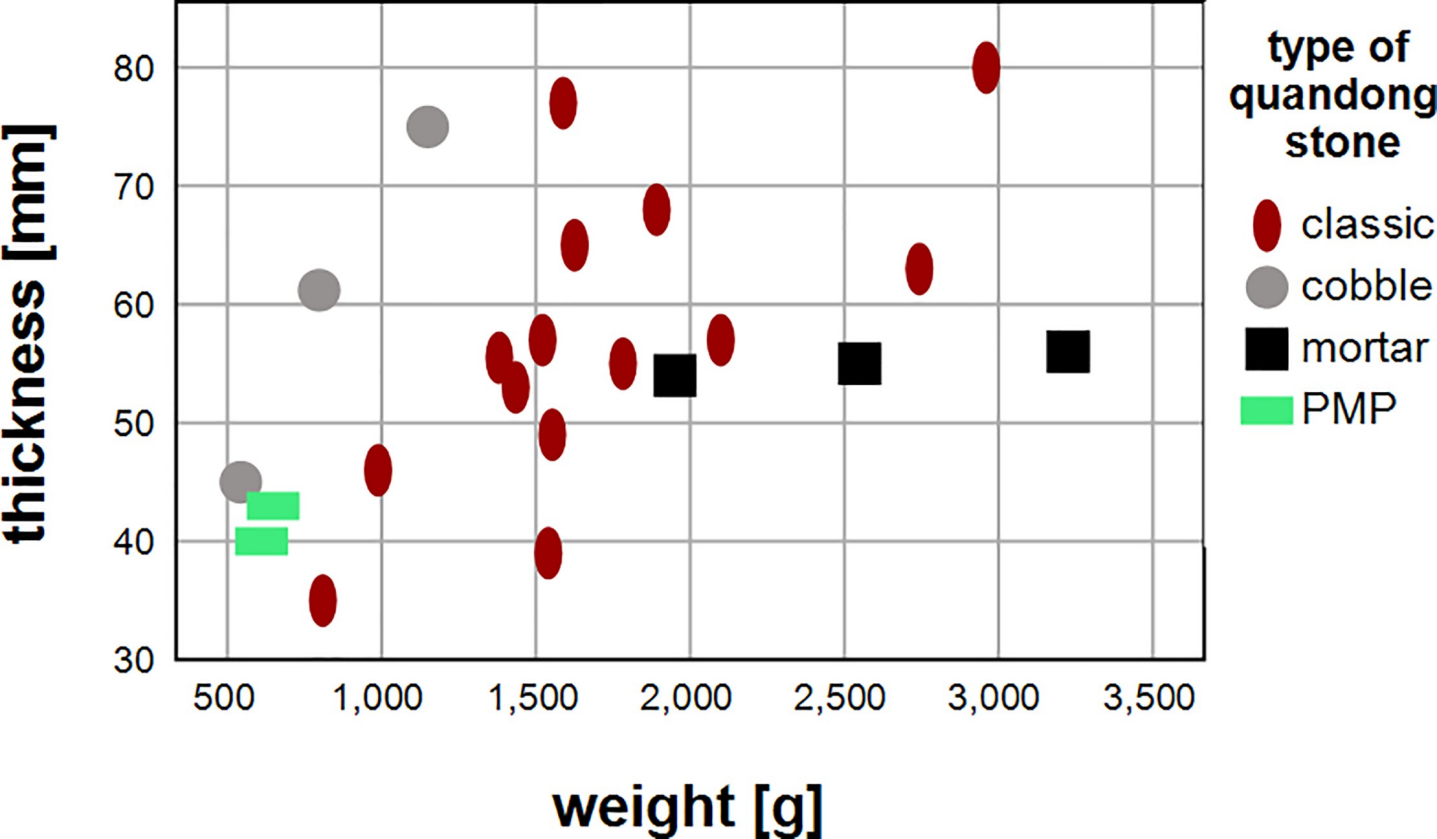
Weight and thickness of quandong stones. The different forms are as identified in the text.

Weights of quandong stones range from 0.5–3 kg although most group around 1.5 kg (S1 Table). Thickness varies between 30 and 80 mm. There is a trend to greater thickness in heavier implements. The mortar-like quandong stones are heavier than the classic form, while PMP-like and amorphous quandong stone implements are less than half the weight (Table 4, Fig 11).

**Table 4:** Average weights for each form of quandong stone.

Form of quandong stone	average weight (g)	n
Classic	1,678.1	13
Amorphous	829.6	3
Mortar	2,199.9	4
PMP	629.7	4
Total	1,532.2	24

### Use-wear and residues

Microscopic analysis of use-wear and residues was not part of the original research design but the potential of such research was discussed with participating Aboriginal groups. One group, West Wyalong Local Aboriginal Land Council, expressed enthusiasm for seeing what results might be achieved from such an analysis. A number of the items they controlled had been collected prior to legislative changes that require a formal government permit for such research. Six ground stone items were provided to Fullagar and Hayes to conduct a pilot study at the Micro Trace Laboratory at the University of Wollongong. All six stones came from the Lachlan River catchment within 50 km of the river channel.

Four of the specimens provided for use-wear and residue analysis had been provisionally identified as quandong stones. These included two of the classic form WW015 and WW019, and two of the mortar form WW050A and 4259 (S1 Table and S1 Fig). Two further stones, not classified as quandong stones, were also analysed. Pestle WW050B had been found adjacent to quandong stone WW050A in a quandong grove suggesting that the two stones may be a functional pair. An additional item 4881, a broken mortar with a shallow facet on each face but no pits, was included (S1 Table and S1 Fig) providing an opportunity to compare use-wear and residues on an artefact not identified as a quandong stone.

Analysis of the items included macroscopic and microscopic examination of both use-wear and residues taking suitable precautions to avoid modern residue contamination–see Supplementary Information for details of laboratory protocols (S1 Text). Interpretation of the results recognised that all stones had been found above ground and had a recent handling history that is not well documented.

Macroscopic manufacture wear included deep pits, likely initiated by hammer. Macroscopic indications of use included ground facets; dished depressions; hammer peck marks; ring cracks; scratch marks; surface texture including sheen or gloss; and plant fibres and rock debris impacted within pits. Microscopic use-wear included fractures on quartz grains, abrasive smoothing, polish, micro-topographic surface levelling and striations. Microscopic residues documented in water extractions consisted of plant tissue (including fibres, starch grains and spores), fungal hyphae and inorganic crystals. Use-wear and residue traces on each of the six MDB items are summarised below and in the Supplementary Information (S1 Fig; S2 Fig; S1 Table; S2 Table; S3 Table).

#### WW015, quandong stone, classic form

**Fig 12:**
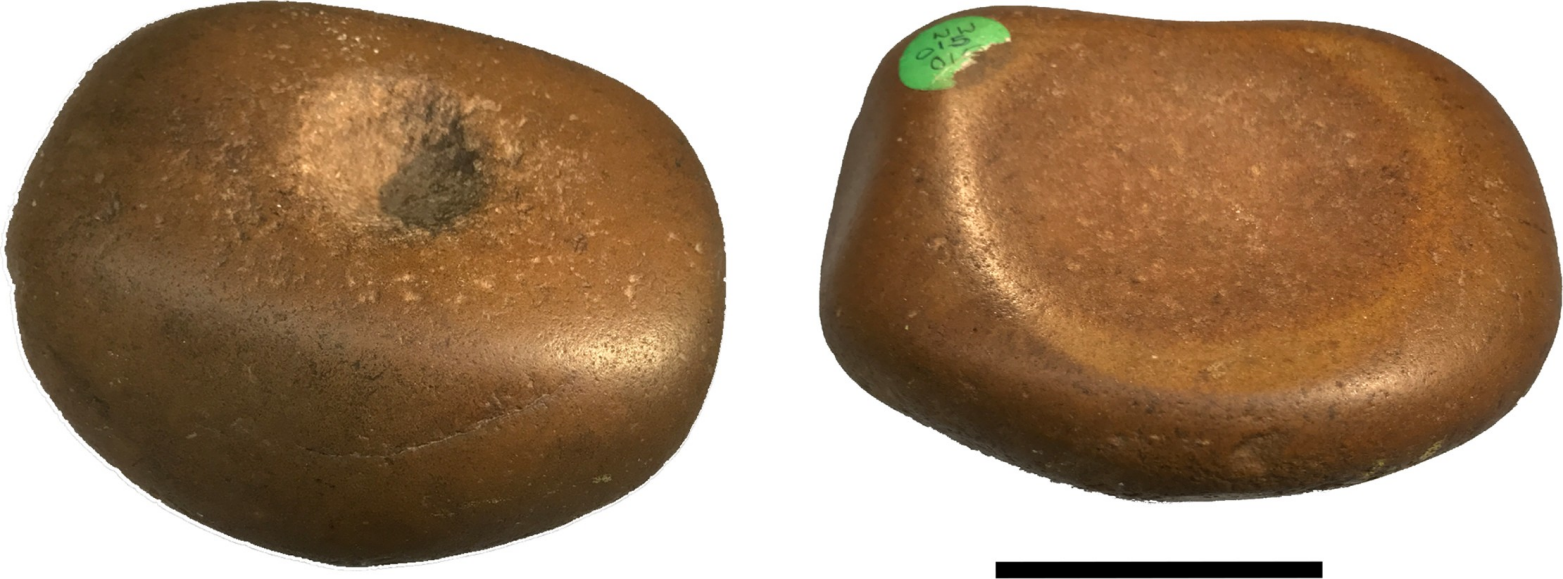
Quandong stone WW015. Left: Surface 1, showing a deep anvil hole and numerous small percussion marks. Right: Surface 2, showing a broad facet, forming a levelled bowl with a smoothed raised rim.

This water-rolled quartzite cobble is a classic quandong stone. It has a single pit on one face and a clearly-defined mortar on the obverse face with a raised, levelled rim (Fig 12; S1 Fig). The stone is highly polished beyond the naturally smooth surface of stream-rolled cobbles (S2 Table) and each face is designed for a distinct activity: nut cracking and kernel grinding that result in different patterns of use-wear. The symmetrical pit (Fig 10) is deep and rounded with a smooth interior surface but without the degree of levelling and polish seen on the mortar surface. There are rare signs of battering in the form of fresh quartz fractures in the pit.

Ring cracks on the upper surface bordering the pit could be the result of miss-hits when cracking nuts, yet even these percussion marks show smoothed and rounded quartz grains, indicating continued smoothing of the top face. The most developed polish zones are on the mortar face and mortar rims (Fig 12; S1 Table; S2 Fig).

Wear traces on quandong stone WW015, such as the formation of use facets–undulating micro-topography with striations and polish on both upper and lower zones–suggest the crushing and grinding of soft plant parts including large seeds and nut kernels typical of diffuse resting percussion in Sophie de Beaune’s conceptual framework [12,41].

Residues were documented under low magnification within the pit on the upper surface of the stone and consisted of crushed rock debris in the form of white granular powder in the lower parts of the micro-topography in addition to smears of granular red/brown tissue. Residues documented in water extractions sampled from within the pit (extraction 1) included plant fibres, amorphous organic material and fungal hyphae. Starch grains were documented in residue extractions sampled from outside of the pit on the upper surface (extraction 2), and included both larger symmetrical grains and asymmetrical grains, ~30 μm in diameter (S2 Fig). Other plant tissues and more hyphae were documented on the lower surface in extractions 3 and 4 (S3 Table). No animal residues were seen.

#### WW019, quandong stone, classic form

**Fig 13:**
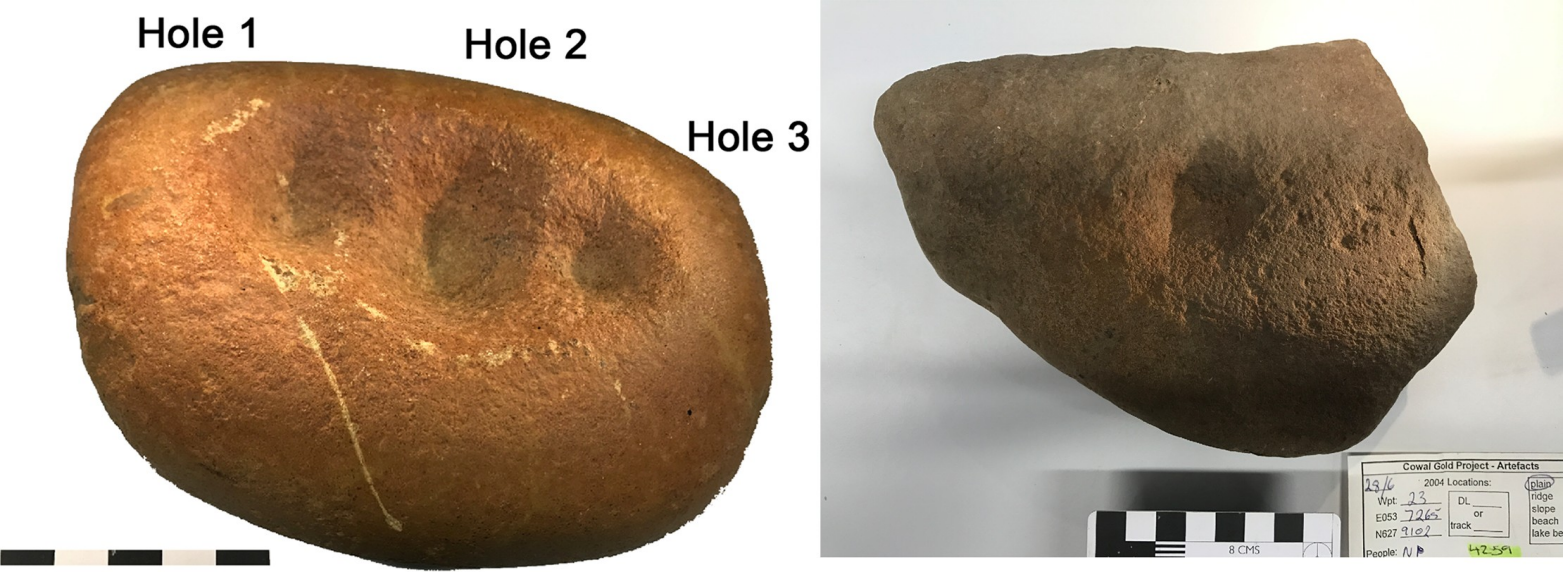
Quandong stones WW019 and 4259. Detail of face with three pits [left]. Fibres were most common in Pit 1; Quandong stone 4259 [right], showing face B. Fibres were found on the PVS peel from the pit.

This water-worn quartzite cobble is another example of a classic quandong stone. It has three deep, rounded, symmetrical pits on one face, and a mortar on the other (Fig 4; S1 Fig). The cobble has a macroscopic gloss all over. Use-wear shows a pattern similar to WW015. Smoothing and polish within pits is mostly on higher microtopography. Polish on the mortar face and rim extends more on lower micro-topography and the surface is more levelled. A few peck marks and fractures are visible on the face surrounding the pits (Fig 13; S2 Table).

**Fig 14:**
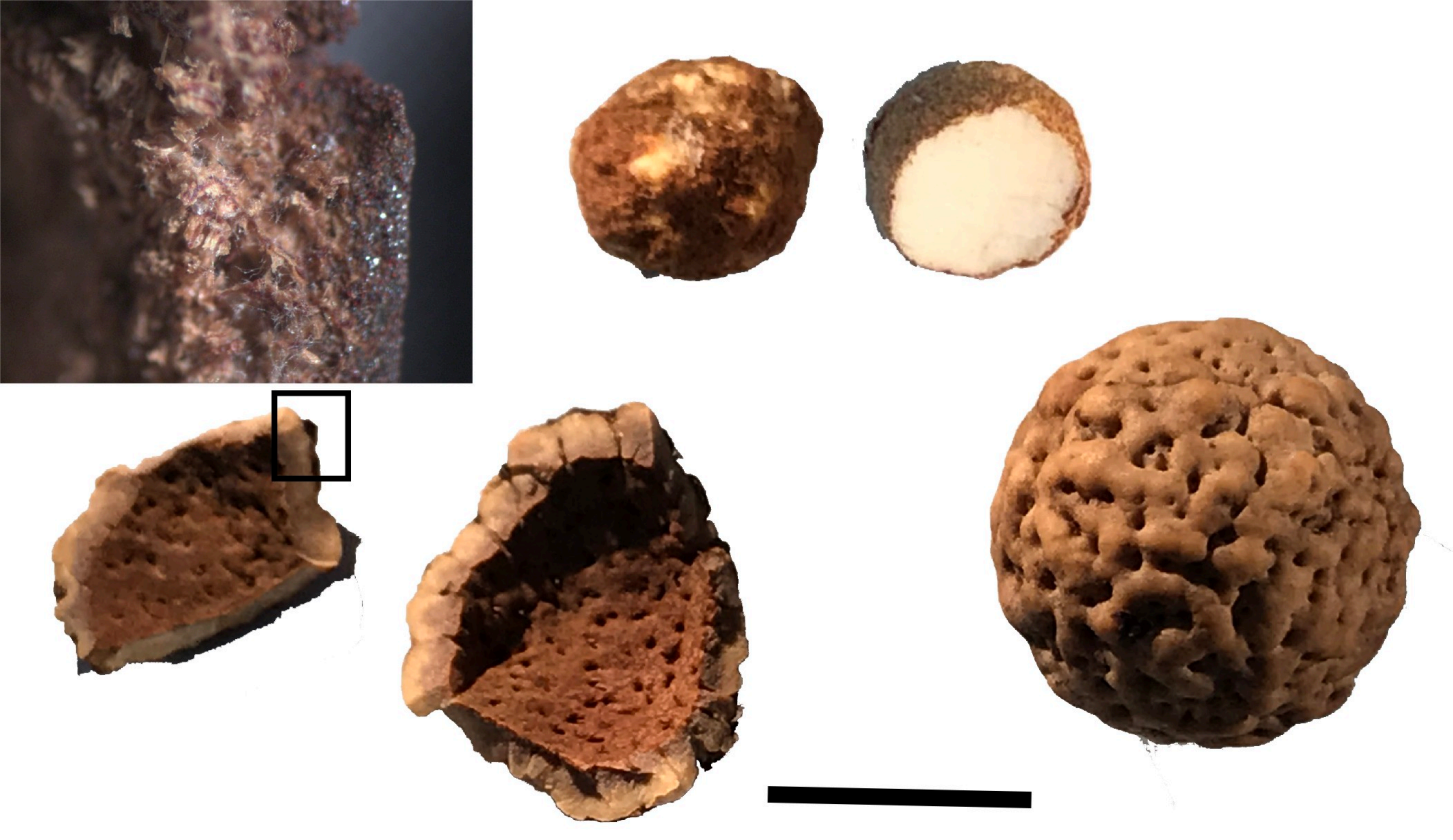
Quandong nut. The broken shell fragments [lower] show the thick, hard outer shell and inner fibrous tissue. The kernel is relatively large with abundant oils [upper]. The scale is in cm.

**Fig 15:**
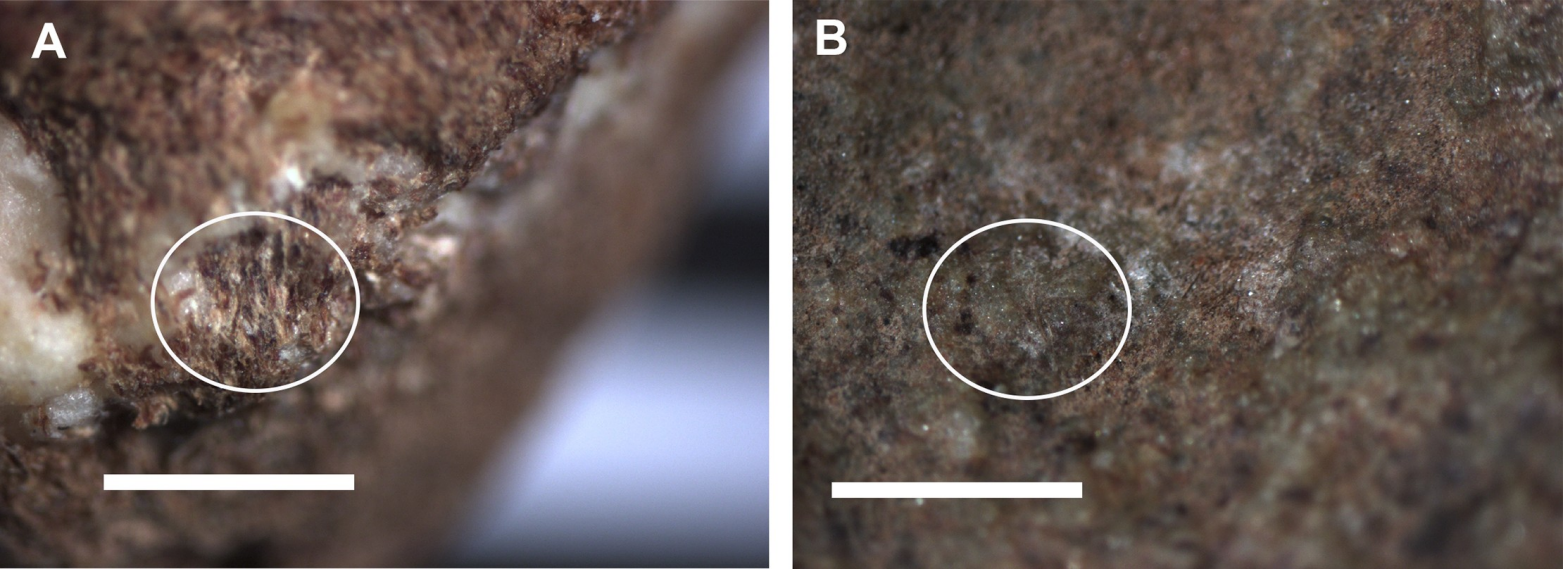
Photo micrographs of fibrous tissue. Broken Quandong nut casing [left, circled]; fibrous matted tissue in situ inside Pit 1 of WW019 [right, circled]. The scale is 1mm.

Quandong nuts collected from the region were examined for residues and included documentation of the outer shell, fibres and kernels (Fig 14). Residues were documented under low magnification within all three pits on the upper surface and included abundant white plant fibres and brown/red residue smears (Fig 15). Compacted fibres extracted from Pit 1 were compared with fibrous tissue from the inner parts of the quandong nutshell and clear similarities of fibre size and form were demonstrated.

Residues documented in water extractions 1, 2 and 3 from each pit consisted of amorphous plant material and other plant residues that were particularly abundant in residue extraction 1 (S2 Fig). Residues were less abundant in water extractions sampled from the mortar face (extractions 4 and 5) and again consisted of fragmented plant tissue (cellulose, fibres and folded tissue). No animal residues were seen.

#### WW050A, quandong stone, mortar form

This water-rolled quartzite cobble is an example of the mortar form of quandong stone. In the classic quandong stones, the nut cracking and grinding functions are on opposing faces of the stone. This item has a ground depression on each face and a single pit in the centre that is rounded and smooth internally. (Fig 7; S1 Fig; S1 Table). The facet in cross section is wider than classic forms and with a different profile, not having as pronounced a shoulder (Fig 10). The stone had been found in a quandong grove alongside a pestle (WW050B). These two items were likely used as a pair.

The cobble has a macroscopic gloss all over, suggestive of continual handling. Microscopic use-wear on the mortar face rim shows micro-topographic levelling but less polish development than in the pits.

Abundant white fibres consisting of small rootlets were documented under low magnification on the upper surface of the stone and are considered to be post-depositional contamination from contact with modern plant tissue.

Other residues potentially relating to use were documented in extractions sampled from within the pits and around the rim on both the upper and lower surfaces. These were most abundant in extractions sampled from the upper surface. Extraction 1 taken from within the pit on the upper surface consisted of abundant amorphous organic material that contained folded plant tissue, including sieve cells and cellulose (S2 Fig). Asymmetrical starch grains ~10–20 μm in diameter were documented in extractions taken from the rim outside the pit on the upper surface (S2 Fig). Some of these starch grains had fissures possibly indicating mechanical damage from hammering. Only a small amount of organic material was seen on the lower surface rim and pit, in addition to bacterial spores and other contaminant residues. Starch was not documented on this lower surface. No animal residues were seen.

#### WW050B, pestle

This pestle, made from a water-rolled quartzite cobble, was found adjacent to quandong stone WW050A. The pestle ends have use-wear most like that on experimental hammers, with uneven freshly fractured grains and hardly any polish development. Use-wear on the sides of the pestle is similar to use-wear found on experimental wood files [51] and shows less polish on lower than upper micro-topography, but more rounding than is found on small, hard seed grinding tools. This polish differs from that caused by weathering and suggests a combination of human handling and working or hitting woody material. (S2 Table; S1 Fig).

Residues were extracted from two sides of the pestle and consisted of amorphous plant tissue in addition to modern plant fibres (S3 Table; S2 Fig). Starch grains were not seen in either extraction. No animal residues were seen.

#### 4259, quandong stone, mortar form

Quandong stone 4259 differs from the classic form. It is not a river cobble but a quartzite slab that has been minimally shaped with smooth, flat facets on both surfaces to form bowl-shaped depressions, each with a deep, rounded central pit. The pits are less clearly defined than those in classic quandong stones. There were few fresh quartzite fractures in the pit on Side B. This item is less highly polished than the classic forms, with little evidence of levelling and abrasive smoothing. The mortar surfaces showed multiple percussion marks consistent with mortar use. Peel PVS 1 taken from a discrete grinding facet on the rim shows an abrasively smoothed polish with no quartz grain fractures as these have been worn down with surface levelling (S2 Table; S1 Fig).

**Fig 16:**
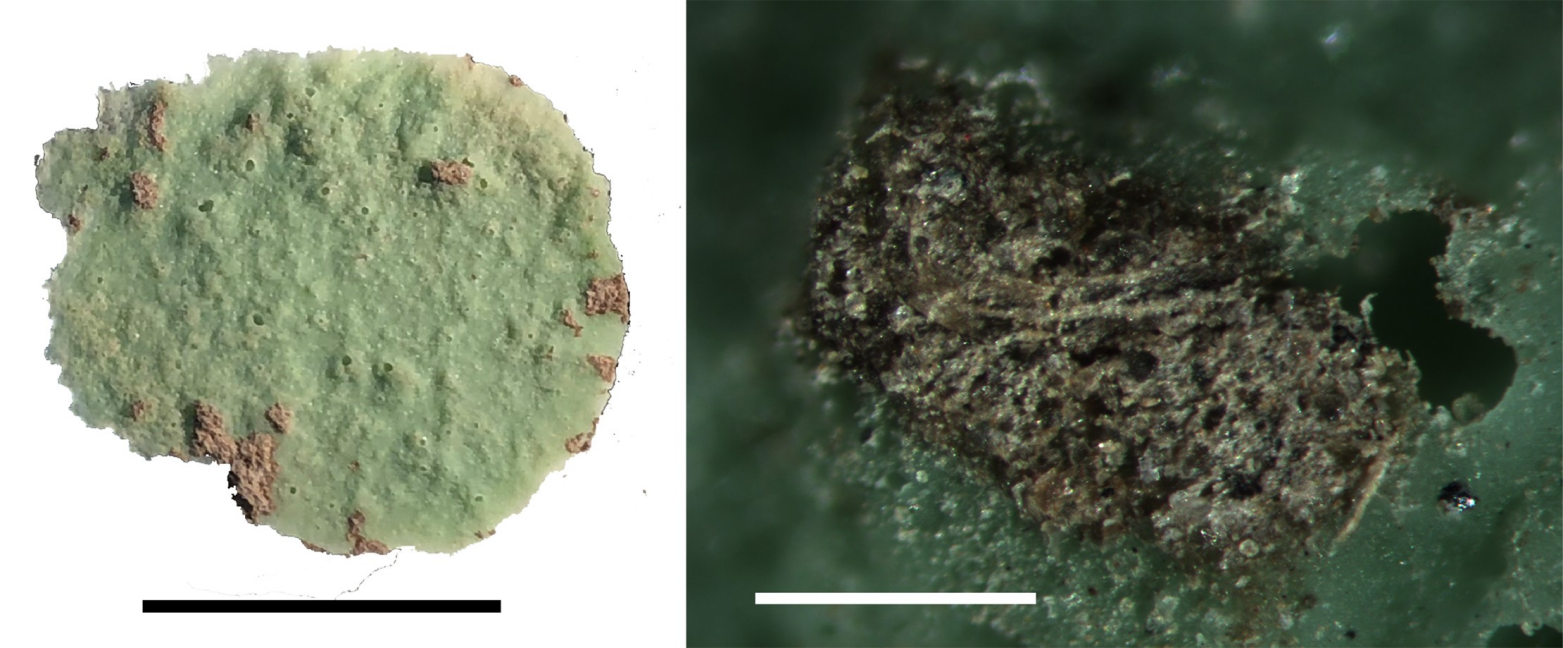
Residues from pit in 4259. A PVS peel from the largest pit in face B of 4259 [left]; fibrous tissue adhered to this peel [right]. The scale is 1mm.

Residues documented in water extractions sampled from both the upper surface (extractions 1 and 2) and the lower surface (extraction 3) consisted of plant residues. No animal residues were seen. Residues were most abundant in extraction 1, sampled from within the pit (S3 Table), and contained asymmetrical, triangular-shaped starch grains approximately 10–20 μm in diameter (S2 Fig). Starch grains were not seen in extractions 2 (rim, upper face) or 3 (rim, lower face), but cellulose fibres were common among sediment residues in extraction 3 (Fig 16; S3 Table).

#### 4881, broken mortar

This small mortar, which is not a quandong stone, was included in this study for comparative purposes. It is made from a stream-rolled quartzite cobble and had been broken in antiquity. It has a moderate depression on both faces. Observed use-wear on this item does not differ significantly from the use-wear on quandong stone mortar surfaces and is characterised by levelling, abrasive smoothing and striations with polish on upper and lower micro-topography.

Residues were abundant in water extractions sampled from both faces of the stone and consisted of plant fibres in addition to round starch grains ~20–30 μm. These starches are larger and more symmetrical than those documented on the quandong stones (S2 Fig). No animal residues were seen. Additionally, haematite crystals were documented on the lower face of the artefact (extraction 2). Although these were few in number, haematite is uncommon in the vertisols of the gilgai plains, but is found in ochre. This item has been subject to in-ground weathering and it is feasible that the observed crystals are a remnant of ochre being ground. This is the only item where haematite residues were observed and the only item not considered to be a quandong stone.

#### Use-wear and residue interpretations

All pits in the quandong stones are consistently deep, rounded and symmetrical with smooth interiors. The use-wear in quandong stone pits is mostly on higher, more-rounded and abrasively smoothed micro-topographic zones and there is little surface levelling. This indicates the absence of stone on stone grinding. There are patches of freshly fractured quartz crystals near the edges and sides of the pits consistent with hard impact. On the most developed zones of polish in pits (WW015) the polish has a slightly reticular pattern similar to use-wear polish on wood-working tools. The same kind of polish on the sides of pestle WW050B is also consistent with the use of the sides for pounding quandong nuts.

The fibres impacted within some of the quandong pits are remarkably similar in form to the fibres on the inner quandong nutshells. These results are preliminary and we plan to undertake experiments with whole quandong fruit to quantitatively assess wear and residues from different parts (nutshell, kernel, and fruit). An initial microscopic analysis of four quandong kernels from nuts collected at Menindee in March 2019 showed abundant oils but no starch grains. This supports previous research that detected only trace amounts of starch in quandong kernels [52]. Starch grains were found in only one pit and no animal residues were found on any of the implements.

The mortar surfaces are generally more levelled than the inside of pits, with patches of polish that extend on both upper and lower micro-topographic zones. The use-wear on some of the mortar depressions suggests processing of soft, oily plant tissue. The polish pattern on these mortar surfaces is most similar to what we see on experimental tools used for crushing or grinding large soft seeds like acorns and water lily seeds and lacks the surface levelling and more reticular pattern we observe on ethnographic and experimental tools used for pounding hard seeds or grinding small softer seeds [53].

Use-wear on the mortar side of Quandong stone WW015 is the most developed and is distinct from the wear on Mortar 4881. The use-wear on other quandong stone mortar surfaces is less developed and overlaps with the use-wear on the grinding surface of Mortar 4881. The high abundance of starch grains on this item contrasts with their rarity in the quandong stone pits and a low abundance on their mortar facets. We interpret this to suggest that quandong stones are used principally for processing nuts and their oily kernels. Mortar 4881 may have been used for processing soft nut kernels, but was not a specialised implement and was likely used to process other starches and possibly ochre.

Previous use-wear studies of pitted stones from Gesher Benot Ya’aqov in the Dead Sea rift [1] have focussed on their potential use for cracking nuts, principally hazelnut (*Corylus spp*). From the palaeobotanical data, seven nut species were seen to co-occur with pitted stones in the same archaeological horizons. Experimental stone knapping using hammer and anvil on similar basalt cobbles produced shallow pits with rough and battered inner surfaces that showed similarities with pits on some of the artefacts found on the site but others ‘were deeper and more rounded with smooth interior surfaces’ [1: 2459]. While recognising that hammering can be used for a variety of functions, the authors argue that ‘only repetitive actions of some duration’ could have led to the distinct wear pattern that they associate with nut cracking pits [1: 2459]. The research concluded that the presence of both battered and smooth interior pit surfaces and various pit shapes on the archaeological items suggested two distinct activities; stone knapping and nut cracking.

Similar questions were addressed at the Mesolithic site of Font del Ros in the Pre-Pyrenees, where pits were seen on a number of cobbles. Experimental cracking, crushing and grinding of roasted hazelnuts did not reproduce a distinctive pattern that would support hazelnut cracking [15]. They attributed the lack of distinct percussion marks to the ‘scanty hardness’ of the hazelnuts and observed that hard-shelled nuts requiring a powerful force to obtain the kernel would produce different results. Our limited experiments with hard-shelled quandong nuts show that they are unlikely to form pits rapidly on the tough quartzite implements that we analysed here. The pits could have been started by hard hammer percussion, becoming wider and deeper with repeated use over many generations.

Experimental research assessing use-wear caused by crushing and grinding hazelnuts (*Corylus*) has described the formation of diagnostic wear traces that include use facets, striations and a glossy sheen. While linked in part to friction action, it is also associated with ‘the lubricating action of the *Corylus* dough which in the zone of greatest friction causes wear that is identified by glossy sheen, and which is associated with the many oily residues produced by crushing and grinding activities’ [3: 1593–4; 12].

Anvil stones used for cracking hazelnuts at the Mesolithic site of Duvensee, northern Germany, ‘possessed a characteristic central depression surrounded by abrasions’ and starch residues associated with nut remains [2: 2873].

Unlike the examples discussed above, our analysis of quandong stones demonstrates a separation of nut cracking and grinding functions. Most of the quandong stones in the MDB collections are of the classic form (13/24). The pits are associated with cracking the nut; the mortar face is used to grind the oily kernels. Based on previous experiments and comparative reference data, the wear and residues in quandong stone pits indicates hard impact and contact with fibrous woody tissue such as nutshells. The polish pattern on the mortar surface and rims indicates the crushing and grinding of large, soft seeds such as quandong kernels.

## Discussion

### Are quandong stones a ‘type’ of implement?

While multi-purpose implements are common across Australia, especially in the arid and semi-arid zones, specialised implements have been recognised. The morah stone, for example, found in a distinct ecosystem in far northern Queensland, has been associated with the grating or grinding of native walnut species [54].

We have demonstrated that quandong stones in the study area are found in areas with a high density of quandong trees and have distinctive macroscopic features. Quandong stones differ from other items with pits. They are often formed from large, flattish river cobbles, and usually have more than one pit, as well as a small mortar bowl on the opposite side. Their pits are deeper and smoother than those on other implements, consistent with efficient nut cracking. This requires specific attributes that do not translate into more generic activities. The preliminary residue and use-wear analyses confirm that hard nuts (with fibrous shells consistent with the quandong) were cracked in the pits.

Quandong nuts are spherical in shape with a very hard shell. A fairly heavy anvil is required with a deep enough pit both to hold the nut in place while being struck and to stop the mallet (axe, hammer or wooden implement) from smashing the nut casing and crushing the kernel. A cobble needs to be large enough to be stable, and tough enough to withstand sustained use during the quandong season.

Multiple pits in quandong stones suggest a high level of activity with large quantities of nuts being cracked at one time. Variation in pit size accommodates the range in nut diameter (Fig 9). Once a sufficient number of kernels have been extracted, the stone would be turned over so that they could be broken up and ground into an oily paste in the smooth mortar dish on the other side.

Studies of hazelnut cracking in prehistoric sites in Europe and the Levant have not distinguished consistently between stones as anvils and stones as hammers when examining either the archaeological record or the results of experimental reconstructions [1,3]. As a result, the published evidence and analyses of use-wear on pits and surfaces are confusing. Some of the other stones with pits in the MDB collections, such as the kulki, hammers and PMPs, may well have been used as top stones for cracking nuts but the resulting pits are smaller with different patterns of use-wear. Since these are portable implements, unlike the quandong stones, we would expect these to be used for a wider range of percussion activities. We are also exploring the idea that a wooden mallet or hitting stick may have been used to crack the nuts [50,55]. The lack of ring cracks or fracture marks on the top of the quandong stones and the polished finish suggests a softer hammer and the siting of the pits in a row along the top of the stones invites speculation that three nuts could have been cracked at once, or in sequence, with such a mallet. We plan to pursue this with ongoing use-wear and residue studies.

### Spatial distribution across the Murray Darling Basin

The larger study from which our interest in quandong stones emerged is spatial in focus and examines the distribution of ground stone implements across the MDB. Intra-site spatial distribution and association of stone implements across individual archaeological sites or cave floors has provided evidence of varied activity areas and functions; the use of seasonal resources, central place foraging strategies; and social organisation [1,15,56]. The scale of our research, and a database that places items within hectares as opposed to centimetres, is very different. It allows us to examine variation, patterning and distribution of implements and archaeological features across a range of ecosystems, to distinguish between long term residence in villages and foraging activities [20,57].

Many of the 1,327 ground stone implements studied are multi-purpose with both grinding and pounding facets and sometimes pits. Such generic tools would have been permanently located in residential sites if heavy, while most people would carry a pair of lighter multi-purpose implements on a daily basis to use for a variety of activities. While the pits in multi-purpose implements may have been used for cracking nuts, they may also have been used for smashing bones for marrow, mincing meat, grinding or mixing ochre for paint, mussel shell and resins for glues or a range of other activities [21,58].

**Fig 17:**
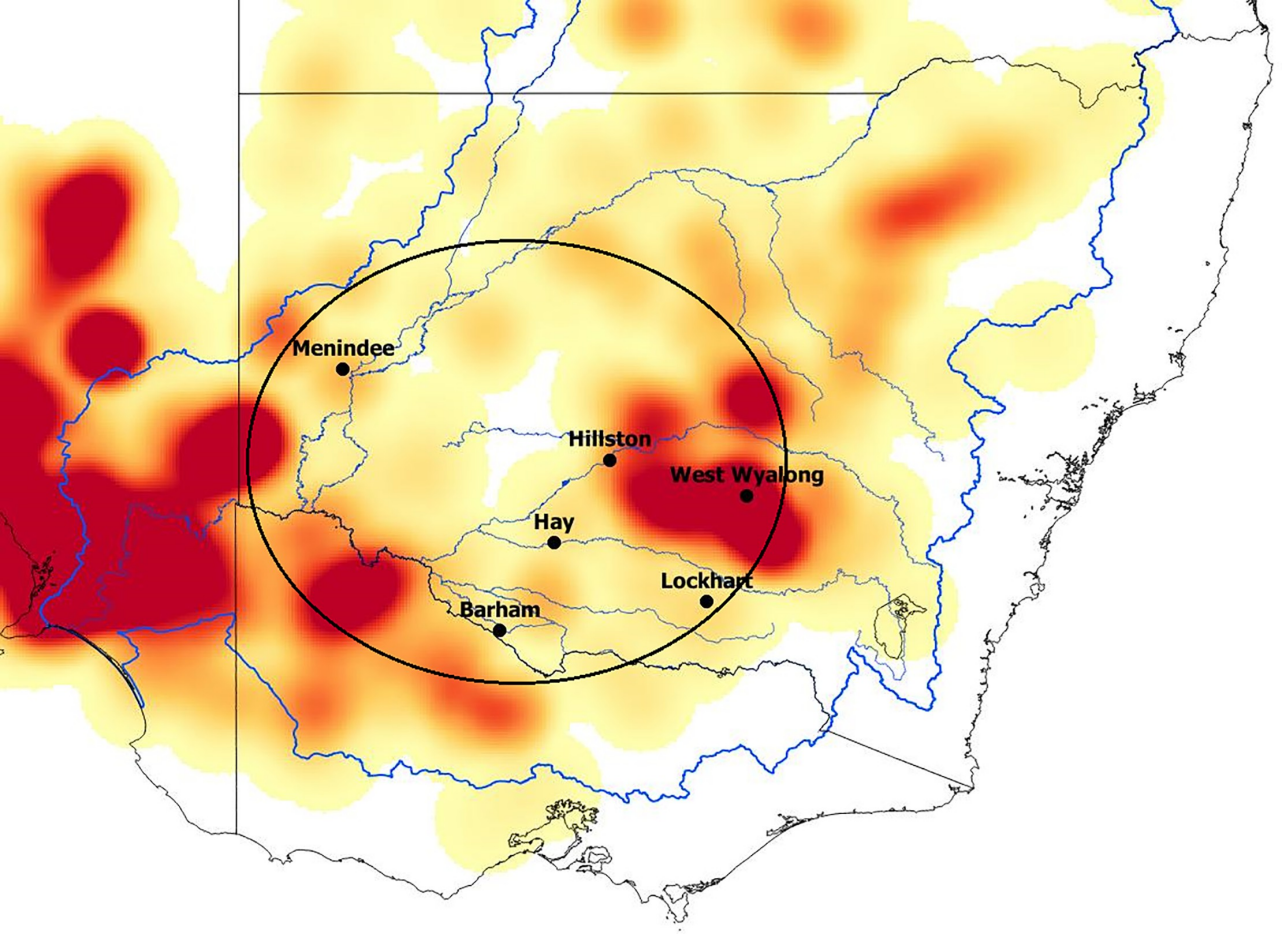
Quandong distribution across the Murray-Darling Basin. The locations of collections that included the quandong stones identified in this study: Menindee, Darling River (n = 3); Barham, Murray River (n = 1), Hay and Lockhart, Murrumbidgee River (n = 7); West Wyalong, Lachlan River (n = 11); Willandra Creek (n = 2). Map created by CP using data from Geoscience Australia (©Commonwealth of Australia (Geoscience Australia) 2012. This material is released under the Creative Commons Attribution 3.0 Australia Licence ‐ http://creativecommons.org/licenses/by/3.0/au/) and Atlas of Living Australia website. Accessed Nov 2018-11-21, https://www.ala.org.au/, ©OpenStreetMap contributors.

The distribution of quandong stones among stone artefact collections in the study area corresponds to the distribution of core areas of quandong trees. The 24 quandong stones were distributed across 51 collections from six localities in five different river catchment areas (Fig 17): the Murray (n = 1), the Murrumbidgee (n = 7), the Lachlan (n = 11), the Willandra (n = 2) and the Darling (n = 3). The collections vary in size and are not evenly distributed across the Basin but only one quandong stone has been recorded among sizeable collections from two areas of the Murray River wetlands. The largest numbers have been documented in collections from the increasingly semi-arid catchments of the Murrumbidgee and Lachlan Rivers, particularly in high-density areas of quandong distribution (Fig 17). The map is compiled from recorded historical occurrences and their occurrence today. European land management practices over 200 years, including land clearance and introduced fauna, have led to a serious decline in quandong numbers today and calls to conserve existing populations to ensure the future survival of the species [59].

Given the nature of the collections, we have little direct evidence linking quandong stones to quandong groves. One of us has the negative evidence of decades of fieldwork across the Basin without encountering a single such a stone in any residential sites. We have verbal evidence from one farmer who produced two very similar stones and when asked where he found them pointed to a grove of quandongs where he had found them lying among the trees.

### Resource procurement strategies

We have sketched out a description of the tools necessary to access the kernel of the quandong, along with a preliminary sampling of their distribution across the central MDB. Objects such as grinding dishes are mainly found in more permanent residences. They are heavy and not well suited to carrying around, weighing on average 8 kg. When seen *in situ*, they are often located close to ground ovens, culturally modified soils, earth mounds and other features typical of longer-term settlement [20]. Dishes are typically found upside down, with the main grinding trough protected from the elements. The muller or top stone will be buried beneath it. Mortars, averaging 2.4 kg, are also heavy enough to consider keeping at home unless, like quandong stones, there is a reason for locating them elsewhere.

If we consider weight as a measure of portability, then it is possible to infer different procurement strategies associated with each type of implement, as documented ethnographically in Arnhem Land [58]. It would only be feasible to test this inference with detailed site locations for each object; identifying whether they occurred in the vicinity of the resource, at a more temporary household, or in a more permanent settlement.

Quandong stones are heavy enough that they become impractical to carry. The fact that they have several pits as well as a mortar bowl on the opposed surface suggests that they may have been kept at particular groves of quandongs, to be used when large numbers of seeds needed to be processed. This large-scale collecting strategy can be contrasted with an occasional expedient foraging strategy, where fortuitous collections of quandongs might be brought back to the settlement to be processed on a household mortar. During periods of greater mobility, people might encounter single quandong trees and could process fewer nuts with the portable mortars or kulki: foraging rather than collecting.

A similar analysis of soft and hard seed grinding implements in a field study of the Menindee Lakes region of the Basin related the distribution of implements to their ecological context [20]. When the distributions of dishes and mortars were compared to a detailed botanical mapping, soft seed grinders were found in areas of densest archaeological record (residential areas typified by culturally modified soils and higher artefact numbers and densities, adjacent to resource zones). Hard seed mortars tended to be distributed among their target resources: in this case acacia species trees associated with more marginal environments. From this we may infer different behavioural strategies. Soft seed grinding dishes indicate a trend to central place collecting and processing, as groups in hamlets or villages. Mortars indicate a foraging strategy of moving to the resources, probably in smaller groups. The correspondence of hard seed mortars with individual ground ovens might suggest smaller, family-sized groups foraging more widely and processing hard seed resources on the spot [20].

Quandong trees are not randomly distributed within Aboriginal territories; they are clustered in groves in specific areas, fruit once a year at a regular time, and are harvested between June and October [60,61]. As one of the few sizable fruits available, Aboriginal people would visit these places on a seasonal basis to harvest the crop over several weeks. While the fruit is readily picked, dried fruit is of greater value as it can be ground or mashed and made into cakes that can be stored and transported long distances. Neither of these activities requires nut cracking but the mortar dish on the obverse face of the anvil would serve this purpose. Once the fruit had been removed, the focus would switch to cracking the nuts to extract the kernels which would be ground into ointment or salve for use through the year or to trade. While people might live close to the productive groves for some weeks at a time during the season, the quandong stones, being heavy, would be cached *in situ* until the following season. Quandong stones indicate a foraging strategy of moving to the resources when in season for large scale processing.

### Chronology

Palaeolithic sites in the Levant give evidence of the importance of nuts in the human diet as far back as 740,000–790,000 years ago [1] with people using stone implements to crack the shell to extract the kernel. Sites in the early Holocene in the Pyrenees and the Netherlands demonstrate the significant role hazel nuts played in the Mesolithic and early Neolithic diet in Europe [2,3].

A study of objects that have principally been collected as a result of erosion or agricultural activities does not lend itself to chronological interpretation, although extensive surface collections provide large sample sizes and regional comparison with plant distributions. We have presented evidence, though, of Aboriginal exploitation of quandongs in historic and prehistoric times using specialised implements. All sandalwood species are endemic to Australia from where they dispersed across the Pacific. *Santalum acuminatum* (the desert quandong) diverged from other species 13 million years ago [62]. Quandongs display low genetic diversity and limited but distinct differentiation between populations [51]. This low genetic differentiation, a poor relationship to geography (lack of geographical structure), and scattered dispersal pattern related to human movement suggests movement of plants mediated by people. The biology of those same people–populations living across the semi-arid and arid zones of the country—follows a similar pattern, one that has been interpreted as the continued extirpation and re-population of the interior from core demographic centres around the margins (the MDB, southwestern WA, the Top End and Cape York) [63].

Archaeological evidence across the continent documents a long series of adaptive changes. Population density and distribution have fluctuated through time in response to climatic changes that have resulted in shifting river systems, desertification, reforestation and changing environmental productivity [64,65]. Resource strategies, economic activities and social organisation will have adapted to these changes over the millennia but the quandong will have been present in the landscape as an important resource for Aboriginal people for the whole of that time. Quandong stones may have changed in form over time, but we do not doubt that they existed in some form.

## Conclusion

We have demonstrated that pits in some ground stone implements are associated with the cracking of quandong nuts in the MDB. While this activity may take place in conjunction with other functions, such as stone knapping on a range of multi-purpose tools, we have provided evidence for pits associated with specialised implements that are used for processing quandongs when in season, both as food source and to produce a salve for medical and skin care purposes. This contributes to an understanding of Aboriginal resource exploitation, highlighting the important role played by quandong fruit and nuts in the daily food quest but also in food storage strategies and possibly trade. The results also provide a methodology for identifying quandong stones in other collections across southern Australia.

Pounding and grinding stones, like ground-edge hatchets, are rarely found in Australian archaeological excavations. However, studies of surface and ethnographic collections provide the opportunity to examine a large number of implements and relate them to plant and stone distributions and cultural practices over large areas, with some temporal resolution provided in particular historical records. Spatial mapping of these implements across different ecological zones can give insights into the patterning of different activities, allowing us to infer different behavioural strategies between sites, across regions, and in response to environmental opportunities and challenges over time.

## Supporting information

S1 Table Quandong stones data table. (XLSX)

S2 Table Forms of use-wear on the pits and facets of analysed MDB grinding stones. (DOCX)

S3 Table Residues from the pits and facets of analysed MDB grindings. (DOCX)

S1 Text Use-wear and residue analysis of MDB grinding stones. (DOCX)

S1 Fig MDB grinding stones analysed for use-wear and residues, showing sampling locations. (TIF)

S2 Fig Micrographs of use-wear and residues on analysed MDB grinding stones. (TIF)
