# Supplementary material for: Quandong stones: A specialised Australian nut-cracking tool
Source: PLoS One. 2019 Oct 2;14(10):e0222680. doi: 10.1371/journal.pone.0222680 (PMC6774476; doi:10.1371/journal.pone.0222680)
Supplement: S2 Table — (DOCX) [file pone.0222680.s002.docx]

# S2 Table. Forms of use-wear on the pits and facets of analysed MDB grinding stones.

|  | **PVS peel location** | **Peck marks** | **Fractures** | **Levelling** | **Abrasive smoothing** | **Polish** | **Striations** |
| --- | --- | --- | --- | --- | --- | --- | --- |
| **WW050A** *quandong stone*   - quartzite cobble - hammer wear - ground surfaces | 1 (pit) | x | few | low | x x | x | x |
|  | 2 (pit rim) | x | x | x | x | low | - |
|  | 3 (pit) | x | few | low | x | x | x |
| **WW050B** *pestle*   - quartzite cobble - ground surfaces | 1 (side) | x | no |  |  |  |  |
|  | 2 (end) | x | few | low | low | low | few |
|  | 3 (end) | x | x | no | low | no | no |
|  | 4 (side) |  | no | x | x | x | x |
| **WW015** *quandong stone*   - quartzite cobble - ground surfaces - hammer wear | 1 (mortar facet) | x | few | x | x | x | x |
|  | 2 (pit) | x | few | x | x | x | x |
|  | 3 (rim, mortar facet) | x | no | x | xx | x | x |
| **WW019** *quandong stone*   - quartzite cobble - ground surface | 1 (mortar facet) |  | no | x | x | x | few |
|  | 2 (pit, rim) | x | few | low | x | x | few |
| **4259** *quandong stone*   - flaked quartzite - ground surfaces | 1 (rim, mortar facet) | x | no | x x | x x | x x | no |
|  | 2 (shallow pit, rim) | x | few | x low | low | x | no |
|  | 3 (pit, rim) | x | x | no | x low | low | few |
| **4881** *mortar*   - quartzite cobble - ground surfaces | 1 (upper mortar facet) | x | no | x | x | x | x |
|  | 2 (lower mortar facet) | x | no | x | x | x | x |

Note: Red text indicates macroscopic and low magnification observations, shaded rows indicate residue sampling from the centre of pits.
