# Supplementary material for: Quandong stones: A specialised Australian nut-cracking tool
Source: PLoS One. 2019 Oct 2;14(10):e0222680. doi: 10.1371/journal.pone.0222680 (PMC6774476; doi:10.1371/journal.pone.0222680)
Supplement: S3 Table — (DOCX) [file pone.0222680.s003.docx]

# S3 Table. Residues from the pits and facets of analysed MDB grindings

|  | **In situ observation** | **Pipette extraction location** | **Starch grains** | **Amorphous organic material** | **Haematite crystals** | **Cellulose** | **Spores** | **Fungal hyphae** |
| --- | --- | --- | --- | --- | --- | --- | --- | --- |
| **WW050A** Quandong stone |  | 1 (pit) | - | x | - | x | - | - |
|  | fibres | 2 (pit margin) | x | x | - | - | - | - |
|  |  | 3 (pit) | - | - | - | - | - | - |
|  |  | 4 (pit rim) | - | x | - | - | x |  |
| **WW050B** Pestle |  | 1 (side) | - | - | - | x* | - | - |
|  |  | 2 (side) | - | x | - | - | - | - |
| **WW015** Quandong stone | crushed rock | 1 (pit) | - | x | - | - | - | x* |
|  |  | 2 (rim) | x | - | - | x | - | - |
|  | fibres | 3 (mortar facet) | - | - | - | x | - | - |
|  | white powder | 4 (rim of mortar facet) | - | - | - | x | - | x* |
| **WW019** Quandong stone | fibres | 1 (pit) | - | x | - | x | - | - |
|  |  | 2 (pit) | - | x | - | - | - | - |
|  |  | 3 (pit) | - | x | - | - | - | - |
|  |  | 4 (pit rim) | - | - | - | x | - | - |
|  | paint* | 5 (mortar facet) |  | fragmented |  | x |  |  |
| **4259** Quandong stone |  | 1 (pit) | x | x | - | - | - | - |
|  |  | 2 (rim) | - | x | - | - | - | - |
|  |  | 3 (lower rim) | - | x | - | x | - | - |
| **4881** Mortar |  | 1 (mortar facet) | x | - | - | x | - | - |
|  |  | 2 (mortar facet) | x | - | x | x* | - | - |

Notes: * indicates probable contamination. Shaded rows indicate residue sampling from the centre of pits.
