# Supplementary material for: Quandong stones: A specialised Australian nut-cracking tool
Source: PLoS One. 2019 Oct 2;14(10):e0222680. doi: 10.1371/journal.pone.0222680 (PMC6774476; doi:10.1371/journal.pone.0222680)
Supplement: S1 Text — (DOCX) [file pone.0222680.s004.docx]

## *S1 Text. Laboratory protocols*

All six ground stone items, including four quandong stones, were examined microscopically using an Olympus SZ61 stereozoom microscope (at magnifications up to x100) with external fibre optic light source and an Olympus BX51 with vertical incident light, at magnifications of x100, x200 and x500. Use-wear was documented within pits and elsewhere on the tool surface using polyvinyl-siloxane (PVS) material. This material provides a high-resolution negative impression of the surface, which can be subsequently examined under vertical incident light to document features such as polish, striations, and micro-fracturing.

Residues were also extracted from all stones by adding up to 40 µL of distilled water to several sampling locations (the ground surface, within pits and the unground surface) using an adjustable pipette fitted with disposable nylon pipette tips. The removed residue mixture (~10 µL of solution) was mounted on a clean glass slide and examined under transmitted light, using the Olympus BX51 microscope. A clean cover slip was placed over the residue mixture and sealed in four corners with clear nail varnish. Suitable precautions (the cleaning of laboratory consumables with ethanol prior to use, the wearing of starch-free gloves, etc.) were taken during analysis to ensure modern residue contamination (including starch) was kept to a minimum. However, the post-depositional context, collection conditions and handling history of the analysed stones are not well documented, Consequently, interpretation of the functional significance of recovered results are considered cautiously.
