# Supplementary figures and images for: Quandong stones: A specialised Australian nut-cracking tool
Source: PLoS One. 2019 Oct 2;14(10):e0222680. doi: 10.1371/journal.pone.0222680 (PMC6774476; doi:10.1371/journal.pone.0222680)

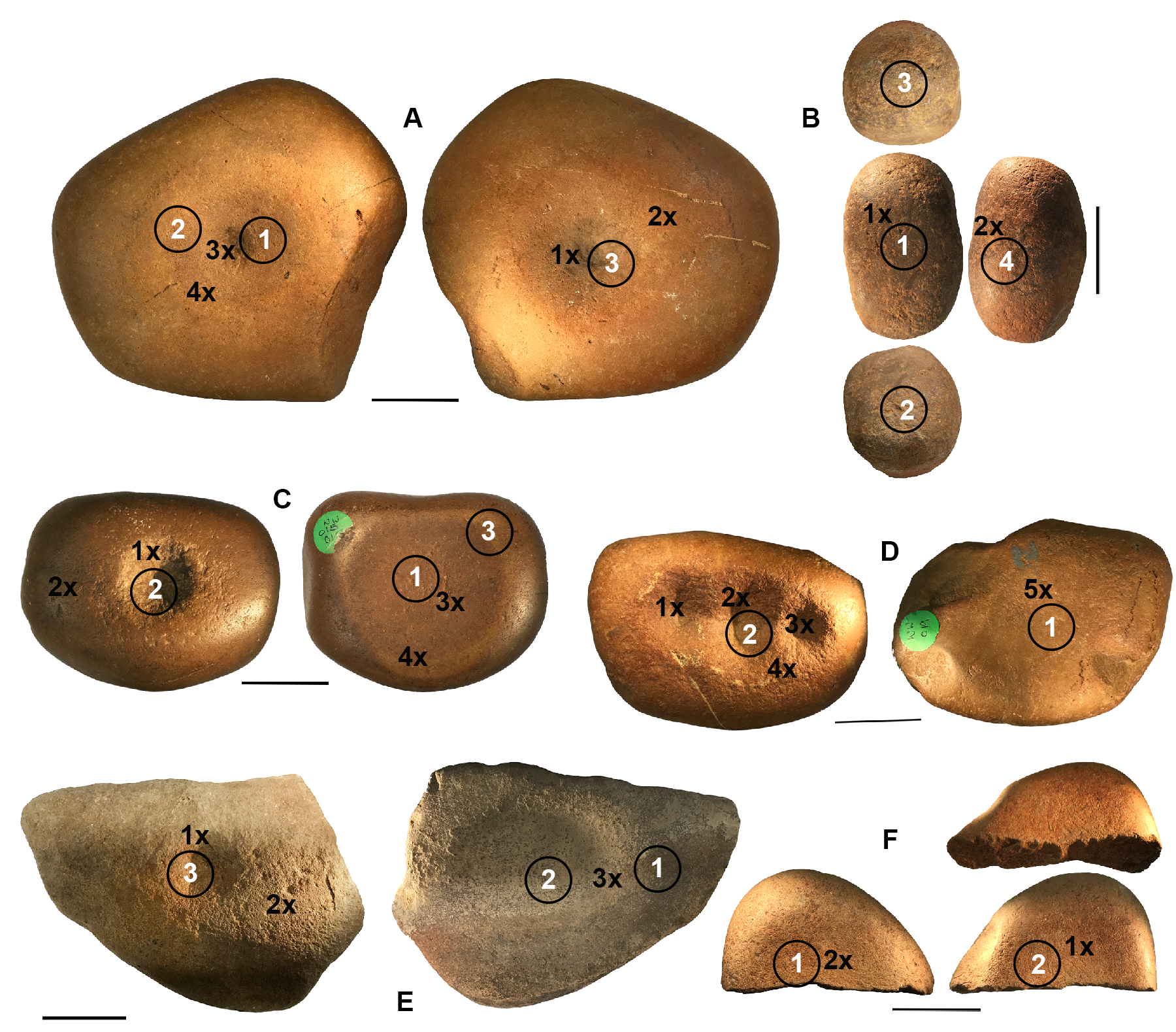

Supplement: S1 Fig — (TIF) [file pone.0222680.s005.tif]

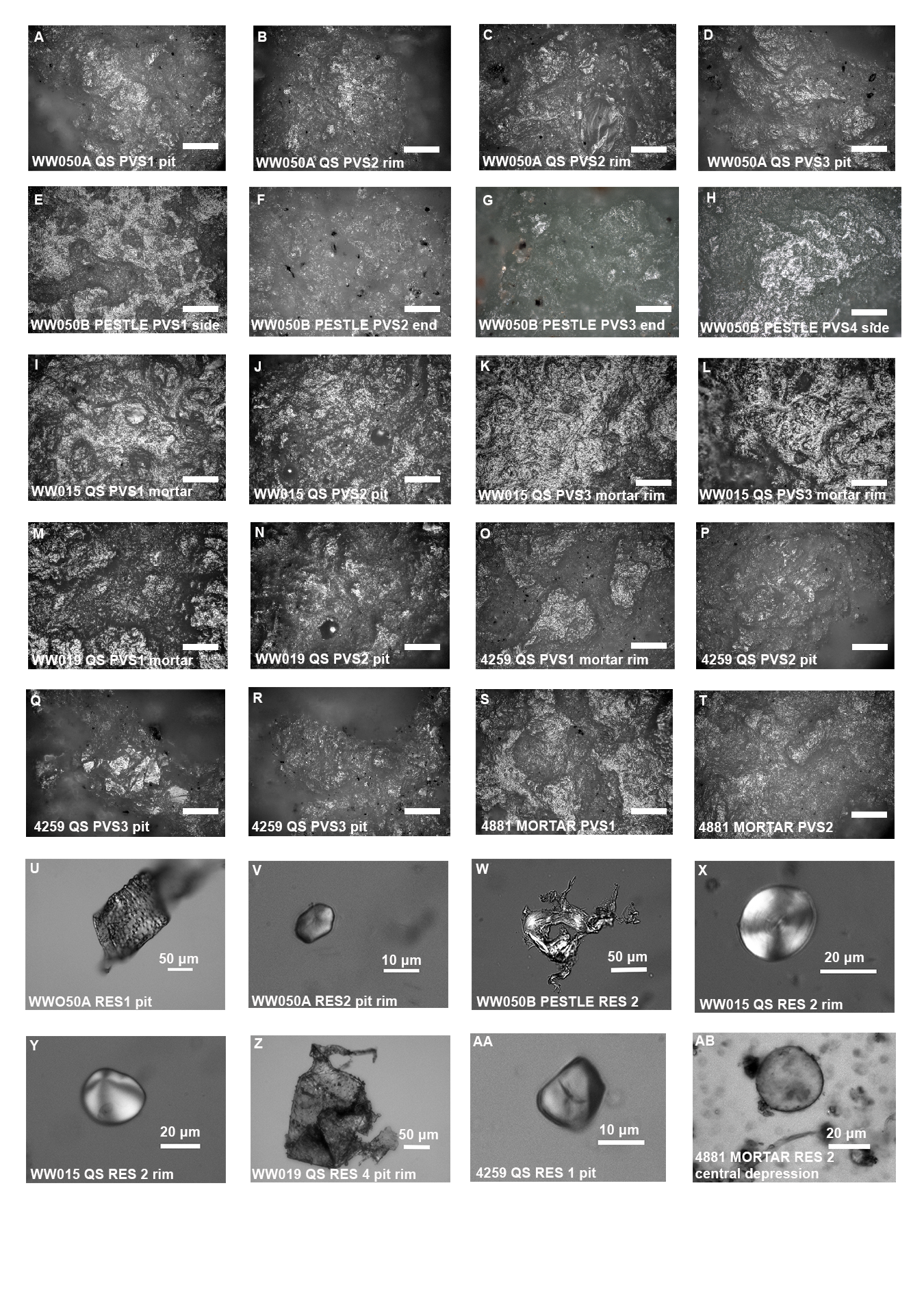

Supplement: S2 Fig — (TIF) [file pone.0222680.s006.tif]
